# The miR-3648/FRAT1-FRAT2/c-Myc negative feedback loop modulates the metastasis and invasion of gastric cancer cells

**DOI:** 10.1038/s41388-022-02451-2

**Published:** 2022-09-24

**Authors:** Weimei Tang, Miaomiao Pei, Jiaying Li, Nanzhu Xu, Wushuang Xiao, Zhen Yu, Jieming Zhang, Linjie Hong, Zheng Guo, Jianjiao Lin, Weiyu Dai, Yizhi Xiao, Xiaosheng Wu, Guangnan Liu, Fachao Zhi, Guoxin Li, Jing Xiong, Ye Chen, Hui Zhang, Li Xiang, Aimin Li, Side Liu, Jide Wang

**Affiliations:** 1 Guangdong Provincial Key Laboratory of Gastroenterology, Department of Gastroenterology, Nanfang Hospital, Southern Medical University, Guangzhou, 510515 China; 2 Department of Hematology and Oncology, International Cancer Center, Shenzhen University General Hospital, Xueyuan AVE 1098, Nanshan District, Shenzhen, Guangdong, 518000 P. R. China; 3 Department of Gastroenterology, Longgang District People’s Hospital, Shenzhen, 518172 China; 4 Department of General Surgery, Nanfang Hospital, Southern Medical University, Guangzhou, 510515 China; 5 Clinical Microecology Center, Shenzhen Hospital, Southern Medical University, Shenzhen, 518000 China; 6 Department of Gastroenterology, Hexian Memorial Affiliated Hospital, Southern Medical University, Guangzhou, 511400 China

**Keywords:** Gastric cancer, Oncogenes

## Abstract

Although the abnormal expression of miRNAs in cancer cells is a widely accepted phenomenon, the molecular mechanisms underlying miR-3648 progression and metastasis in gastric cancer (GC) remain unclear. miR-3648 expression is downregulated and its ectopic expression in GC cells significantly suppressed cell proliferation and metastasis. Mechanistic analyses indicated that miR-3648 directly targets FRAT1 or FRAT2 and inhibits FRAT1- or FRAT2-mediated invasion and motility in vitro and in vivo. Moreover, FRAT1 physically interacted with FRAT2. Furthermore, FRAT1 overexpression promoted GC cell invasion, whereas siRNA-mediated repression of FRAT2 in FRAT1-overexpressing GC cells reversed its invasive potential. Besides, miR-3648 inactivated the Wnt/β-catenin signalling pathway by downregulating FRAT1 and FRAT2 in GC. Interestingly, c-Myc, a downstream effector of Wnt/β-catenin signalling, was also downregulated by miR-3648 overexpression. In turn, c-Myc negatively regulated miR-3648 expression by binding to the miR-3648 promoter. In addition, miR-3648 expression levels were negatively correlated with c-Myc, FRAT1, and FRAT2 expression in fresh gastric samples. Our studies suggest that miR-3648 acts as a tumour-suppressive miRNA and that the miR-3648/FRAT1-FRAT2/c-Myc negative feedback loop could be a critical regulator of GC progression.

## Background

Gastric cancer (GC) is the fifth most common cancer and the third most common cause of cancer-related deaths worldwide [1]. Despite recent progress in the detection and treatment of early GC, the long-term survival rate of patients with advanced GC is low [2]. Thus, identification of diagnostic biomarkers and a better understanding of the molecular mechanisms underlying GC progression are urgently needed.

MicroRNAs (miRNAs or miRs), approximately 22 nucleotides in length, are believed to regulate gene expression by binding to the 3′-untranslated region (3′-UTR) of mRNAs, thereby leading to mRNA degradation or blocking of mRNA translation [3, 4]. Various miRNAs have been reported to be involved in GC progression. For instance, ectopic expression of miR-647 significantly inhibited SRF mRNA and protein and miR-647 expression was negatively associated with SRF mRNA [3]. Our previous reports verified that miR‐646 is a tumour suppressor for GC that directly regulates the expression of FOXK1 [4].

miR-3648 is a less well-studied miRNA. It is located on chromosome 21p11.2 [5] and is involved in several types of cancer. Reports have shown that miR-3648 modulates prostate cancer cell proliferation by targeting APC2 [6]. In addition, overexpression of miR-3648 regulates bladder cancer cell migration and invasion [7]. However, the biological function and underlying mechanisms of miR-3648 in GC remain unclear.

The Wnt/β-catenin signalling pathway is one of the most important intracellular pathways that control cancer progression [8]. Wnt/β-catenin signalling is transduced through the Frizzled family of receptors and the LRP5/LRP6 co-receptor to the β-catenin signalling cascade [9, 10]. PAR-1, CKI epsilon, FRAT1, and FRAT2 are positive regulators of the canonical Wnt pathway, whereas APC, AXIN1, AXIN2, and PPARg are negative regulators [9, 11]. The nuclear complex, consisting of TCF/LEF [12], activates the transcription of canonical Wnt target genes, such as c-Myc [13], JUN [14, 15], cyclin D1 [16, 17], and HIF-1a [18]. Some reports show that miRNAs regulate target genes in the Wnt/β-catenin signalling pathway. For example, miRNA-194 activates the Wnt/β-catenin signalling pathway in GC by targeting SUFU [19]. miR-504 suppresses the mesenchymal phenotype of tumours by directly targeting the FZD7-mediated Wnt-β-catenin pathway [20]. However, whether miR-3648 participates in the Wnt signalling pathway in GC is not fully understood.

Here, we present evidence that miR-3648 is a potential tumour suppressor that directly targets FRAT1-FRAT2 (frequently rearranged in advanced T-cell lymphomas-1 and 2) to inactivate the Wnt/β-catenin pathway. Furthermore, we observed that miR-3648 was inhibited by an oncogenic transcription factor, c-Myc–a Wnt/β-catenin-downstream target gene, thus forming a negative feedback loop among miR-3648/FRAT1-FRAT2/c-Myc, thereby enhancing GC progression.

## Results

### Downregulation of miR-3648 expression is associated with poor prognosis in GC

We first performed a genome-wide screen to identify the miRNAs associated with the clinical outcomes of patients with GC according to strict statistical criteria (*P* < 0.01) in the TCGA database using oncomir online resources (http://www.oncomir.org/). We found that 26 miRNAs were associated with overall survival (OS).

**Fig. 1:**
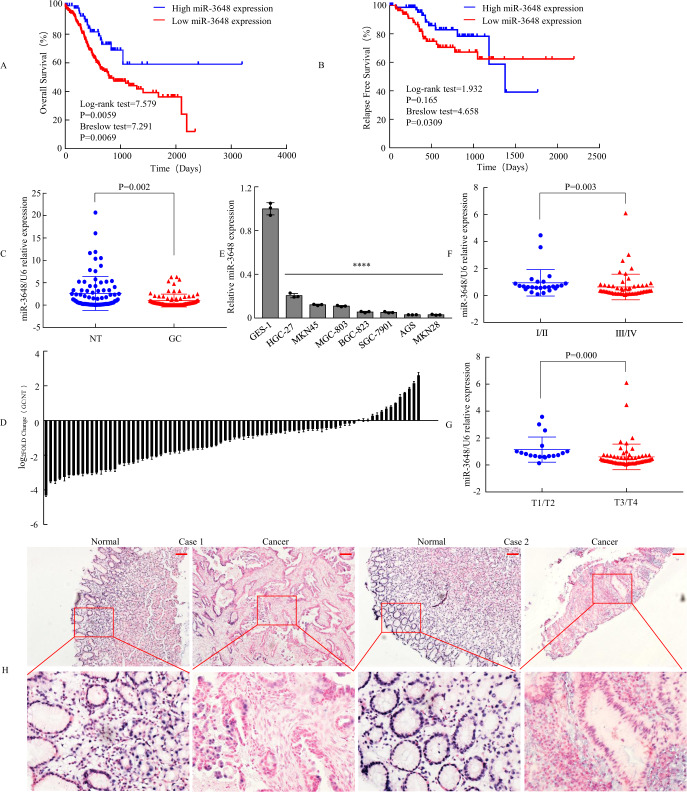
miR-3648 expression was associated with prognosis and downregulated in GC. **A**, **B** Survival analyses of overall survival (OS) and relapse-free survival (RFS) were performed based on Kaplan–Meier curves in oncomir database. **C** Expression of miR-3648 was determined by qPCR in 82 human gastric tissues, which was normalised against an endogenous U6 RNA control. NT normal mucosal tissues, GC GC tissue. ***P < 0.01, paired *t*-test. **D** Relative miR-3648 levels of GC and normal tissues measured by qPCR were shown using a waterfall plot. Error bars represent the mean ± SD from three independent experiments. **E** miR-3648 expressions in eight gastric epithelial cell lines by qPCR. One-way ANOVA and Dunnett’s T3 multiple comparison test. ****P < 0.001 between normal and cancer gastric epithelial cells. The result is presented as the mean ± SD of three independent experiments. **F**, **G** The relationship between miR-3648 expression and clinical stages or local invasion. ***P < 0.01; ****P < 0.001. *P* values were estimated using two-tailed unpaired Student’s *t*-test. **H** ISH analysis of miR-3648 expression level in normal gastric mucosa and GC tissues. Scale bars, 100 μm in **H**.

The high expression of 15 miRNAs predicts poor prognosis and acts as a potential oncogene in patients with GC (Supplementary Table 2), whereas the low expression of 11 miRNAs predicts unfavourable prognosis and functions as a putative tumour suppressor (Supplementary Table 3). Among them, expression of miR-3648 was associated with both short-term and long-term OS of patients (Fig. 1A). Moreover, expression of miR-3648 was associated with short-term relapse-free survival of patients, but expression of miR-3648 was not associated with relapse-free survival over time (Fig. 1B).

Second, we focused on the low expression of 11 miRNAs: miR-1306-3p, miR-3648, miR-2115-3p, let-7f-5p [21], miR-7-5p [22], miR-96-3p, miR-942-5p [23], miR-135b-3p, miR-423-5p [24], miR-221-3p [25], miR-15b-5p [26]. Using the PubMed-MEDLINE database, we found that miR-1306-3p, miR-2115-3p, miR-96-3p, miR-135b-3p and miR-3648 have not yet been reported in GC. We detected the expression of these five miRNAs in 30 human gastric tissues using qPCR. Except for the increased expression of miR-135-3p, the expressions of the other four miRNAs were decreased in GC tissues (T) than in normal gastric mucosa (N) (Supplementary Fig. 1). Among them, miR-3648 was the most profoundly downregulated. Thus, we wondered whether the deregulation of miR-3648 might be involved in tumour development and progression. We then measured miR-3648 expression in 82 gastric specimens and verified the results of the above-mentioned studies (*P* = 0.002, Fig. 1C, D).

Third, we compared the levels of miR-3648 in seven GC cell lines and the normal gastric cell line GES-1. Result showed that miR-3648 levels were lower in all GC cells compared to GES-1 cells (*P* < 0.001, Fig. 1E). This was consistent with our clinical findings that human gastric tissues have low levels of miR-3648 (Fig. 1C, D). In particular, miR-3648 expression levels significantly decreased in GC tissue samples from patients with advanced cancer (stage III + IV) compared with GC tissue samples from patients with early-stage cancer (stage I + II) (Fig. 1F). Fourth, miR-3648 expression was lower in deeper invasion GC (T3–4 vs. T1–2), suggesting that its deficiency may contribute to GC cell invasiveness (Fig. 1G). Fifth, we performed ISH of gastric samples. The results also showed that the expression of miR-3648 in GC was significantly lower than that in the normal gastric mucosa (Fig. 1H and Supplementary Fig. 2)

Taken together, these findings suggest that miR-3648 is downregulated in GC tissues and can be implicated as a tumour suppressor miRNA in GC.

### Exogenous miR-3648 suppresses the malignant biological behaviour of GC cells in vitro

To evaluate the clinical correlation between miR-3648 expression levels and clinicopathological characteristics in patients with GC, we investigated the relative expression levels of miR-3648 in 82 GC tissue samples. We showed that the low expression of miR-3648 was correlated with differentiation (*P* < 0.05), depth of invasion (*P* < 0.001), lymph node metastasis (present vs. absent, *P* = 0.001), and TNM stage (I–II vs. III–IV, *P* < 0.001), but was not correlated with patients’ age, sex, and tumour size (*P* > 0.05). These studies suggest that low-level expression of miR-3648 is associated with malignant progression in patients with GC (Supplementary Table 4).

**Fig. 2:**
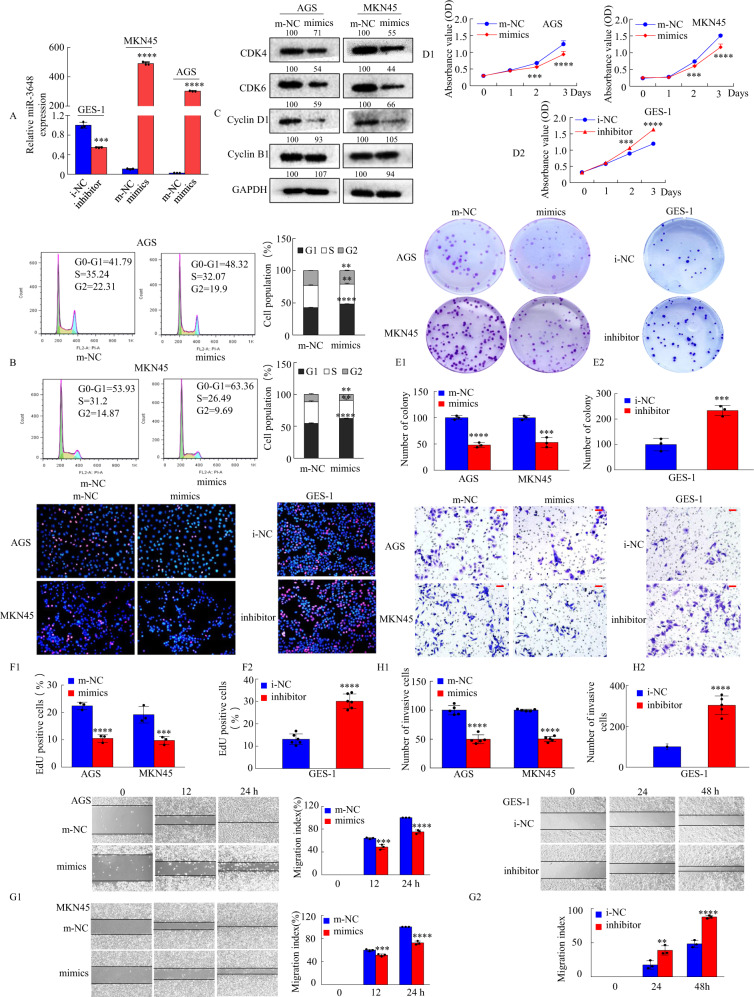
miR-3648 modulates biological functions of GC cells in vitro. **A** The GC cells transfected with miR-3648 mimics or miR-3648 inhibitors at 48 h and the expression of miR-3648 was evaluated by qRT-PCR. ***P < 0.01; ****P < 0.001. Error bars represent the mean ± SD from three independent experiments. *P* values were estimated using two-tailed unpaired Student’s *t*-test. **B** The cell cycle phases of the treated cells were evaluated by flow cytometry. The percentage of each phase is presented. **P < 0.05; ****P < 0.001, two-tailed unpaired Student’s *t*-test. **C** Whole-cell lysates of GC cells were prepared, and protein expression was detected by western blotting. The values of the grey scale were labelled near the bands. **D**–**F** The effect of miR-3648 mimics or inhibitors on cell proliferation was evaluated by CCK-8 (**D**), colony formation (**E**) and EdU (5-ethynyl-2´-deoxyuridine) (**F**) assay. ***P < 0.01 and ****P < 0.001; m-NC vs. mimics and i-NC vs. inhibitor. **G1**, **G2** Wound-healing assays were used to detect GC cell motility following transfection with mimics or inhibitors. Wound closure percentages are shown on the right or under the panel. ***P < 0.01 and ****P < 0.001, m-NC vs. mimics; **P < 0.05 and ****P < 0.001, i-NC vs. inhibitor. **H1**, **H2** Invasion assays were conducted using cells transfected with miR-3648. Quantitative results are shown under the panel. ****P < 0.001, m-NC vs. mimics; ****P < 0.001, i-NC vs. inhibitor. Scale bars, 50 μm in **F** and **H**. Error bars represent the mean ± SD. All experiments were repeated at least three times with identical findings. *P* values were estimated using two-tailed unpaired Student’s *t*-test.

To detect the role of miR-3648 expression in cell growth and metastasis in GC cells, we transfected miR-3648 mimics or m-NC into AGS and MKN45 cells or miR-3648 inhibitor or i-NC into GES-1 cells, confirming miR-3648 expression via qRT-PCR (Fig. 2A). Next, we examined whether miR-3648 is involved in the proliferation of GC cells. Flow cytometry analysis showed that the overexpression of miR-3648 resulted in an increased number of cells in the G0/G1 phase of the cell cycle (Fig. 2B). Moreover, expression of cell cycle-related proteins, such as cyclin D1, CDK4, and CDK6, significantly decreased upon treatment with miR-3648 mimics, whereas cyclin B1 levels remained unchanged compared with m-NC-treated AGS and MKN45 cells (Fig. 2C). Furthermore, CCK-8 assays showed that treatment with miR-3648 mimics resulted in significantly lower optical density values at 450 nm, whereas the transfection of GES-1 cells with the miR-3648 inhibitor resulted in higher OD values than the corresponding NC group (Fig. 2D1, D2). Colony formation assays showed much lower colony formation in the miR-3648 mimic group than in the m-NC group. Conversely, transfection of GES-1 cells with a miR-3648 inhibitor had the opposite effect (Fig. 2E1, E2). In addition, EdU assays revealed similar results (Fig. 2F1, F2).

The effect of miR-3648 on cell motility and invasion was tested using scratch wound-healing and invasion assays. The results showed that ectopic miR-3648 expression significantly inhibited the migration and invasion ability of AGS and MGN45 cells, whereas treatment with a miR-3648 inhibitor increased migration and invasion capacity of GES-1 cells (Fig. 2G1, G2, H1, H2).

Taken together, these in vitro results suggest that miR-3648 suppresses GC cell growth, migration, and invasion.

### miR-3648 directly targets the FRAT1 and FRAT2 3′-UTR

**Fig. 3:**
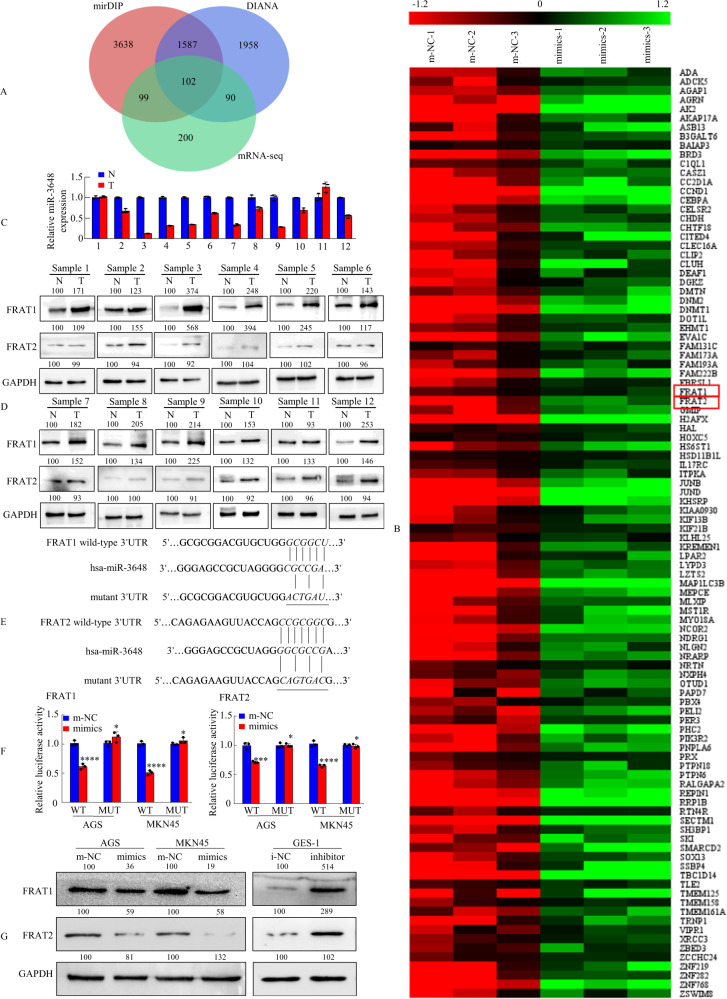
miR-3648 directly targets FRAT1 or FRAT2 in GC cells. **A** The three-way Venn diagram indicated the numbers of genes that overlapped in two publicly available bioinformatics algorithms (mirDIP and DIANA) and RNA sequencing-based miR-3648 signature. **B** The heatmap was based on 102 candidate genes that were downregulated in AGS cells. Red colour represents an expression level above mean, green colour represents an expression lower than the mean. Highlighted by red box. **C** miR-3648 level and **D** FRAT1/FRAT2 protein expression levels in twelve freshly collected gastric mucosal tissues using qRT-PCR and western blot analyses. Error bars represent the mean ± SD from three independent experiments. **E** Putative miR-3648-binding sequence within the 3′-UTR of FRAT1 or FRAT2 mRNA. Mutations in the complementary site for the seed region of miR-3648 in the 3′-UTR of the FRAT1 or FRAT2 gene are indicated. **F** Luciferase reporter vectors containing wild-type (wt) or mutant (mut) FRAT1 or FRAT2 3′-UTR were constructed and cotransfected with miR-3648 mimics into AGS and MKN45 cells. Luciferase reporter assays were used to determine whether miR-3648 directly binds to the 3′-UTR of FRAT1 or FRAT2. *P > 0.05, ***P < 0.01 and ****P < 0.001, m-NC vs. mimics. Error bars represent the mean ± SD from three independent experiments. *P* values were estimated using two-tailed unpaired Student’s *t*-test. **G** Western blot analysis of FRAT1 or FRAT2 levels in gastric epithelial cells treated with miR-3648 mimic or inhibitor.

To delineate the molecular mechanisms by which miR-3648 regulates GC cell growth and metastasis, two publicly available bioinformatic algorithms (mirDIP and DIANA) and RNA sequencing-based miR-3648 signatures were used to analyse the target genes of miR-3648. The collection of genes with absolute fold change >1.2 in AGS cells was taken and intersected with the results from two published target prediction engines, mirDIP and DIANA. The results revealed that 102 genes were downregulated in miR-3648-overexpressing cells compared to m-NC cells (Fig. 3A, B). Enrichment analysis of these 102 genes was conducted in Metascape (https://metascape.org/). The most significant GO Biological Process was the Wnt signalling pathway, which was enriched with a total of 12 genes including CCND1, CELSR2, MST1R, SKI, TLE2, FRAT1, FRAT2, KREMEN1, ZBED3, LZTS2, NRARP and NLGN2 (Supplementary Fig. 3A). Among these candidates, CCND1 [27], MST1R [28], SKI [29], FRAT1 and FRAT2 [30] have been reported to act as oncogenes in GC. Thus, we carried out qPCR to detect the mRNA expression of CCND1, MST1R, SKI, FRAT1 and FRAT2 after increasing or inhibiting the expression of miR-3648. As shown in Supplementary Fig. 3B, C, FRAT1 and FRAT2 have the most obvious change. Thence, FRAT1 and FRAT2 were identified as potential targets.

To identify the correlation between miR-3648 and the expression of potential target genes FRAT1 or FRAT2, we investigated miR-3648 and FRAT1 or FRAT2 expression in 12 pairs of human GC tissues and matched normal gastric mucosa via miRNA and western blot analyses. As shown in Fig. 3C, D, most patients exhibited lower miR-3648 levels but higher FRAT1 and FRAT2 protein levels in cancer tissues (T) compared to normal gastric mucosa (N). Spearman’s correlation analyses (Supplementary Fig. 3D) revealed a negative correlation between miR-3648 and FRAT1 (Supplementary Fig. 3D1) and between miR-3648 and FRAT2 (Supplementary Fig. 3D2), and a positive correlation between FRAT1 and FRAT2 (Supplementary Fig. 3E) expression.

We performed a luciferase reporter assay to determine whether FRAT1 or FRAT2 is indeed regulated by miR-3648. To accomplish this, we constructed 3′-UTR reporter plasmids encoding full-length wild-type (wt) or mutant (mut) miR-3648 binding sites for FRAT1 or FRAT2 (Fig. 3E). We found that miR-3648 repressed the reporter activity of the wt-FRAT1-3′-UTR or wt-FRAT2-3′-UTR plasmid but not that of the mut-FRAT1-3′-UTR or mut-FRAT2-3′-UTR plasmid (Fig. 3F).

Next, western blot analysis showed that the overexpression of miR-3648 suppressed endogenous FRAT1 or FRAT2 expression in AGS and MKN45 cells, whereas blocking miR-3648 expression resulted in the upregulation of endogenous FRAT1 or FRAT2 expression in GES-1 cells (Fig. 3G).

Taken together, these results suggest that miR-3648 can downregulate FRAT1 and FRAT2 expression by directly targeting its 3′-UTR.

### FRAT1 and FRAT2 are involved in the miR-3648-mediated inhibition of GC cell invasion and metastasis

**Fig. 4:**
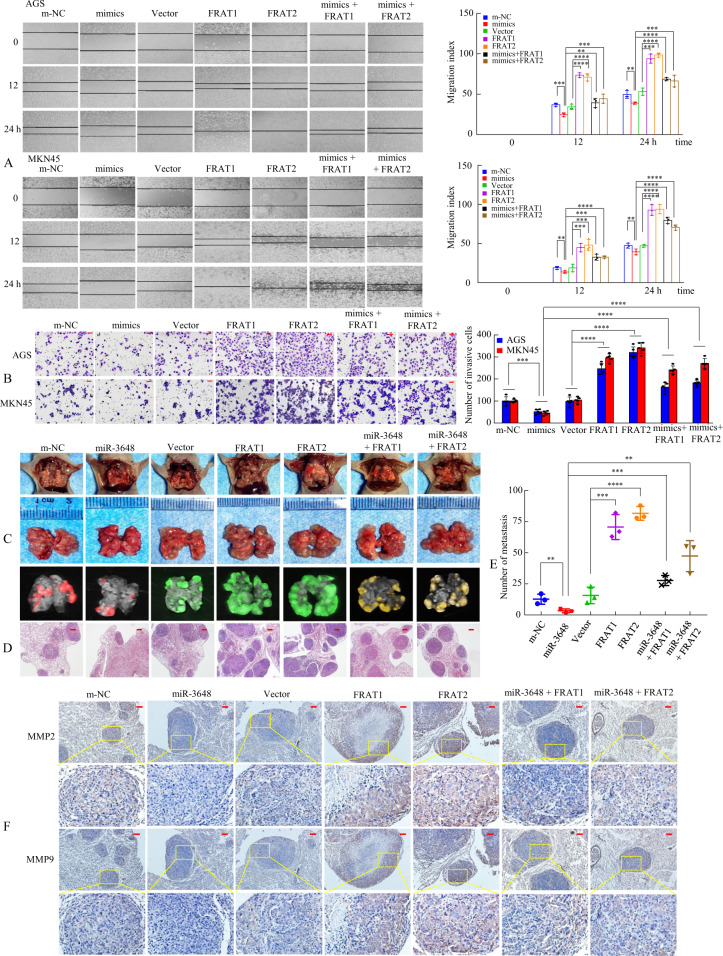
FRAT1 or FRAT2 mediate the effects of miR-3648 on tumour invasion and metastasis in GC cells. **A** The migration ability and **B** the invasion ability of cells transfected with miR-3648 mimics and/or FRAT1 or FRAT2 expression plasmid were determined using a wound-healing or the transwell assay. **P < 0.05; ***P < 0.01; ****P < 0.001. The result is presented as the mean ± SD. *P* values were estimated using two**-**tailed unpaired Student’s *t*-test. All experiments were repeated at least three times with identical findings. **C** The GC MKN45 cells were orthotopically transplanted into the lung of nude mice and mice were randomly divided into seven groups as follows: m-NC, miR-3648, Vector, FRAT1, FTAT2, miR-3648 + FRAT1 and miR-3648 + FRAT1 group (*n* = 3 in each group, one of three nude mice was showed). Error bars represent the mean ± SD. *P* values were estimated using two**-**tailed unpaired Student’s *t*-test. **D** The mice were sacrificed and metastatic cancer tissues were stained with haematoxylin and eosin (H&E). **E** Representative images of metastatic loci in the lungs were shown. Number of metastatic loci in the lungs was counted. **P < 0.05; ***P < 0.01; ****P < 0.001. **F** Immunohistochemical (IHC) staining of MMP2 and MMP9 expression. Scale bars, 50 μm in **B**, 200 μm in **D** and 100 μm in **F**.

FRAT1 and FRAT2 are important effectors of some miRNAs in human cancers [31–33]; therefore, there is a need to further explore the biological effects of miR-3648 on FRAT1 and FRAT2. We transfected AGS and MKN45 cells with m-NC, miR-3648 mimics, vector, FRAT1 plasmid, or FRAT2 plasmid and cotransfected them with miR-3648 + FRAT1 or miR-3648 + FRAT2. Cell function assays showed that upregulation of miR-3648 resulted in lower proliferation, migration, and invasion capacity, whereas ectopic upregulation of FRAT1 or FRAT2 plus miR-3648 partially reversed the influence of miR-3648 on GC cell growth, migration, and invasion compared with the control cells (Supplementary Fig. 4A, B and Fig. 4A, B).

Previous reports showed that FRAT1 or FRAT2 is related to cancer cell invasion and motility. Thus, we determined whether FRAT1 or FRAT2 is involved in the miR-3648-mediated inhibition of GC cell metastasis in vivo. The MKN45 cells transfected with Lv-m-NC, Lv-miR-3648, Lv-Vector, Lv-FRAT1, Lv-FRAT2, Lv-miR-3648 + FRAT1, or Lv-miR-3648 + FRAT2 were injected into the tail vein of nude mice (Fig. 4C and Supplementary Fig. 4C). Histological analysis of H&E stained lung sections verified the existence of lung metastases (Fig. 4D). We showed that the number of metastatic loci was obviously reduced in mice injected with miR-3648-expressing cells compared to those injected with m-NC cells, whereas FRAT1 or FRAT2-expressing cells formed more metastatic nodules than FRAT1/miR-3648 or FRAT1/miR-3648 co-expressing and vector-expressing cells (Fig. 4E). MMP2 and MMP9 are gelatinases of matrix metalloproteinase family, which were initially associated with the invasive and metastatic properties of tumour cells, owing to their ability to degrade major protein components of the extracellular matrix and basement membranes [34]. Thus, immunohistochemical (IHC) staining using anti-MMP2 and anti-MMP9 antibodies further verified the metastatic ability of the different groups (Fig. 4F). Collectively, our data demonstrate that miR-3648 suppresses the invasion and metastasis of GC by downregulating FRAT1 or FRAT2.

### Interaction of FRAT1 and FRAT2 promotes invasion and metastasis in GC cells

The FRAT1 and FRAT2 genes, which are cancer-associated genes, are clustered in the human chromosome at the 10q24 locus [30]. Therefore, we first investigated whether a correlation exists between FRAT1 and FRAT2 expression in GC cells. We investigated the interacting proteins of FRAT1 in the GeneMANIA database (http://genemania.org/) and the results showed that FRAT1 protein maybe binds to FRAT2 protein. Pearson correlation analyses showed positive correlations between FRAT1 and FRAT2 using the GEPIA database (http://gepia.cancer-pku.cn/) (Supplementary Fig. 5A). We then examined the expression of FRAT1 and FRAT2 in 82 human GC tissues and found positive correlations between FRAT1 and FRAT2 via qPCR (Supplementary Fig. 5B).

**Fig. 5:**
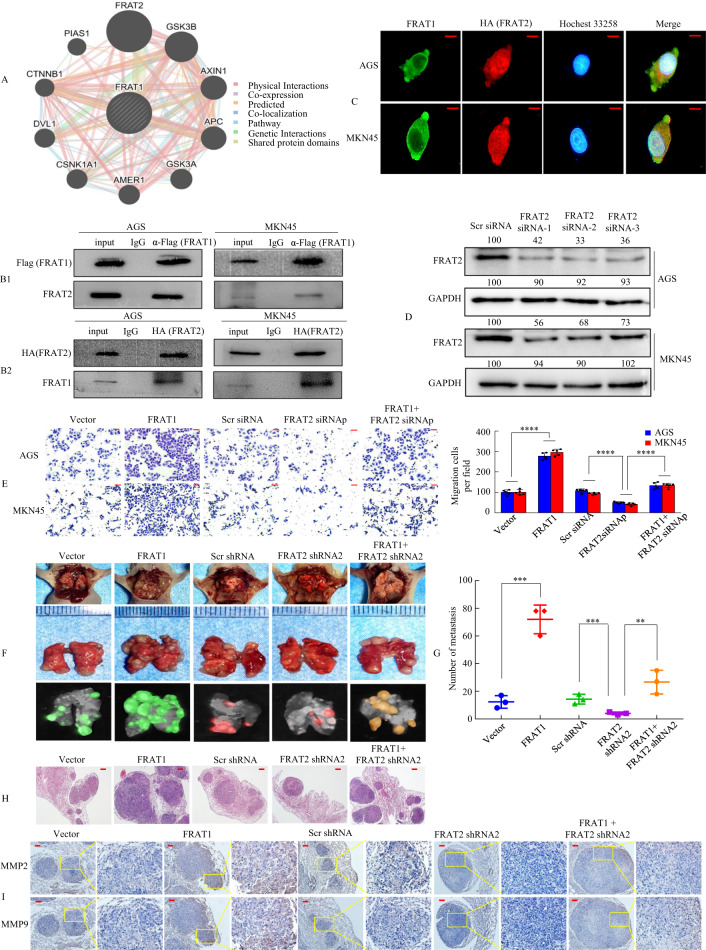
FRAT1 synergises with FRAT2 to promote tumour invasion and metastasis. **A** Potential FRAT2 binding partners were predicted using the GeneMANIA database. The red boxes represent protein–protein interactions. **B1**, **B2** Interaction between FRAT1 and FRAT2 in GC cells. Flag-tagged FRAT1 (**B1**) or HA-tagged FRAT2 (**B2**) plasmid was transfected into cells. Immunoprecipitation was performed with Flag-tagged FRAT1 or HA-tagged FRAT2 antibody, and pre-immune normal mouse immunoglobulin G (nm IgG) was used as a control. Western blot analysis was performed with an anti-FRAT2 (α-FRAT2) or anti-FRAT1 (α-FRAT1) antibody. **C** Double staining of FRAT1 (green) and HA-FRAT2 (red) in GC cells was visualised by confocal microscopy; nuclei were counterstained with Hoechst 33258. **D** The protein levels of FRAT2 in AGS and MKN45 cells with four treatments [Scrambled (Scr) siRNA, FRAT2 siRNA 1, FRAT2 siRNA 2 and FRAT2 siRNA 3] were determined by western blot analysis. **E** In vitro the invasive ability of AGS and MKN45 cells were evaluated by Tranwell assay. siRNAp, siRNApool. ****P < 0.001. Error bars represent the mean ± SD. *P* values were estimated using two-tailed unpaired Student’s *t*-test. **F** Mice were orthotopically transplanted with MKN45 cells (*n* = 3 in each group, one of three nude mice was shown). Error bars represent the mean ± SD. *P* values were estimated using two-tailed unpaired Student’s *t*-test. **G** Metastatic cancer tissues were stained with H&E. **H** The number of metastatic loci in the lungs was counted. **P < 0.05; ***P < 0.01. **I** IHC staining of MMP2 and MMP9 expression. The scale bars represent 20 μm in **C**, 50 μm in **E**, 200 μm in **G** and 100 μm in **I**.

To further verify the physical interaction between FRAT1 and FRAT2 in vitro, we transfected Flag-tagged FRAT1 into AGS and MKN45 cells and assessed whether the FRAT1 protein interacts with FRAT2. We showed that an anti-Flag (FRAT1) antibody could co-immunoprecipitate FRAT2 from cell extracts (Fig. 5B1). Similarly, an anti-HA (FRAT2) antibody could co-immunoprecipitate FRAT1 (Fig. 5B2). Confocal fluorescence microscopy showed that the co-localisation of the two proteins was apparent in the merged images (Fig. 5C).

We synthesised three pairs of FRAT2 siRNAs in GC cells and confirmed them via western blotting (Fig. 5D). To show whether FRAT1 synergises with FRAT2 during metastasis and invasion, GC cell invasion in vitro was determined using scratch wound-healing and transwell assays. We showed that the forced expression of FRAT1 increased the migration and invasion capacity compared to treatment with the vector alone, whereas FRAT2 downregulation in FRAT1-overexpressing cells decreased the cell motility and invasion potential of FRAT-overexpressing cells (Supplementary Fig. 5C and Fig. 5E).

To further confirm that FRAT1 synergises with FRAT2, we also synthesised three FRAT1 siRNAs. The effects of these three FRAT1 siRNAs were confirmed by western blot (Supplementary Fig. 6A). We assessed the migration and invasion capabilities of GC cells using wound-healing assays and transwell invasion assays. We found that depressing the expression of FRAT1 or FRAT2 respectively could inhibit the migration and invasion ability of GC cells, while depressing the expression of FRAT1 and FRAT2 simultaneously enhanced the inhibitory effect significantly (Supplementary Fig. 6B, C).

To address the functional consequences of FRAT1 cooperating with FRAT2 on tumour metastasis, we inoculated MKN45 cells transduced with Lv-vector, Lv-FRAT1, Lv-shRNA, Lv-FRAT2 shRNA2, or Lv-FRAT1 + FRAT2 shRNA2 into BALB/c-nu/nu mice (Fig. 5F and Supplementary Fig. 6D). The presence of GC metastasis in the lung was confirmed via histological analysis (Fig. 5G). Larger lung metastatic nodules were discovered in the Lv-FRAT1 group than in the Lv-vector group. In contrast, inoculation with Lv-FRAT1-FRAT2-shRNA2 cells reversed the effects observed in mice inoculated with Lv-FRAT1 cells (Fig. 5H). IHC assays showed that the overexpression of FRAT1 resulted in a significant upregulation of the metastatic markers MMP2 and MMP9, whereas the loss of FRAT2 in FRAT1-overexpressing cells caused a decrease in MMP2 and MMP9 expression (Fig. 5I).

Taken together, these findings suggest that the FRAT1-FRAT2 axis contributes to cancer progression and metastasis.

### miR-3648 inhibits GC cell metastasis through the inactivation of the Wnt/β-catenin signalling pathway by downregulating FRAT1/FRAT2

**Fig. 6:**
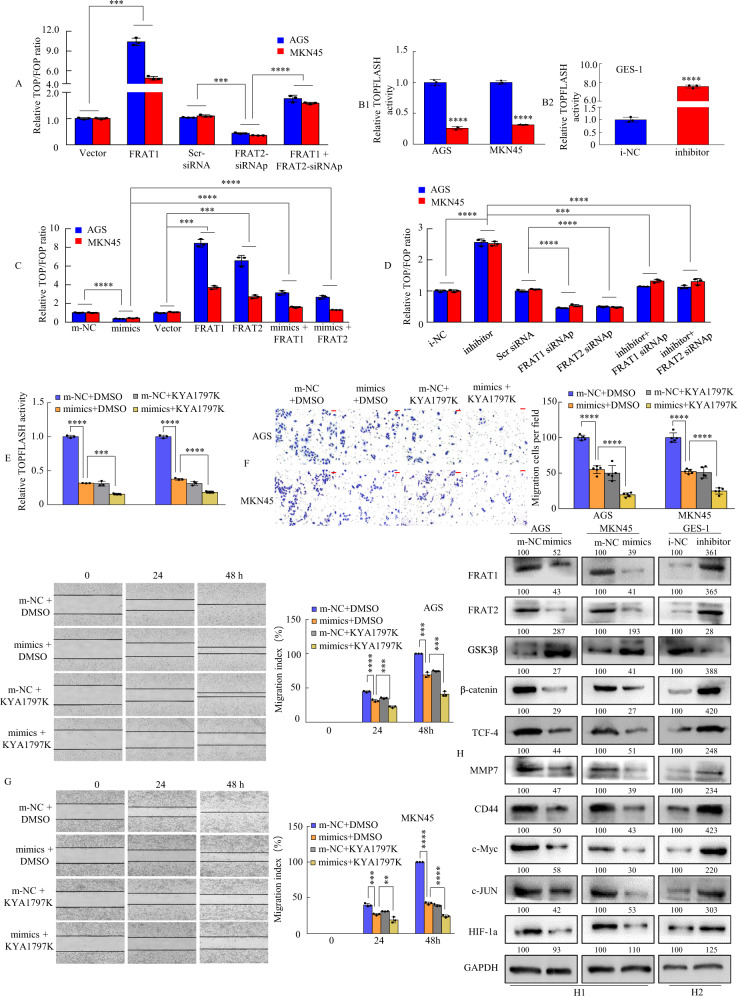
miR-3648 suppressed the activation of Wnt-β-catenin signalling by targeting FRAT1/2 in vitro. **A** The GC cells were cotransfected with Vector, or FRAT1, or Scr siRNA, or FRAT2 siRNAp, or FRAT1 + FRAT2 siRNAp and TOP/FOPflash reporter plasmid were measured 48 h, and the luciferase activity was determined. Normalised to Renilla luciferase activity used as an internal control. ***P < 0.01; ****P < 0.001. Two-tailed unpaired Student’s *t*-test. **B1**, **B2** miR-3648-modulated GC cells were transfected with the TOP/FOPflash reporter plasmid, and the reporter activities were determined 48 h. ****P < 0.001; ****P < 0.001. Two-tailed unpaired Student’s *t*-test. **C** The GC cells were cotransfected with m‐NC, or miR‐3648, or Vector, or FRAT1, or FRAT2, or miR‐3648 + FRAT1, or miR‐3648 + FRAT2 and TOP/FOPflash reporter plasmid. Then, Luciferase activity was tested 48 h later. ***P < 0.01; ****P < 0.001. Two-tailed unpaired Student’s *t*-test. **D** The GC cells were cotransfected, and the luciferase activity was measured after 48 h. Normalised Renilla luciferase activity was used as an internal control. ***P < 0.01; ****P < 0.001. Two-tailed unpaired Student’s *t*-test. **E** The GC cells were cotransfected with 100 ng of TopFlash or FOPFlash luciferase reporter and m-NC + DMSO, or miR-3648 + DMSO, or m-NC + KYA1797K, or miR-3648 + KYA1797K, then treated for 48 h. Luciferase activity was measured. ***P < 0.01; ****P < 0.001. Two-tailed unpaired Student’s *t*-test. **F**, **G** Effects of m-NC + DMSO, or miR-3648 + DMSO, m-NC + KYA1797K, or miR-3648 + KYA1797K on GC cells migration and invasion using transwell and wound-healing assay. **P < 0.05; ***P < 0.01; ****P < 0.001. Two-tailed unpaired Student’s *t*-test. All experiments were repeated at least three times. **H1**, **H2** Western blot analysis of ten protein expressions in GC cells transfected with miR-3648 mimics or miR-3648 inhibitor. Cells transfected with control (NC) plasmids were used as a control. The densitometry data presented below the bands are fold change compared with control. Scale bars, 50 μm in **F**.

Previous studies have revealed that FRAT1 and FRAT2 are important members of the Wnt/β-catenin signalling transduction pathway [35–38]. Therefore, we first transiently cotransfected GC cells with a FRAT1 vector and/or FRAT2 siRNA and pTOPFLASH or pFOPFLASH reporter plasmids. The TOPFLASH/FOPFLASH reporter assay is a relative quantitative detection method to measure Wnt/β-catenin signalling pathway activity [39, 40] and was performed 48 h post-treatment in GC cells. We found that ectopic expression of FRAT1 significantly enhanced β‐catenin‐dependent luciferase activity, whereas FRAT2 downregulation in FRAT1-overexpressing cells reversed the enhanced effect (Fig. 6A). Second, we tested whether miR-3648 regulates the Wnt/β-catenin signalling pathway. The results showed that miR-3648 is a negative regulator of Wnt/β-catenin signalling (Fig. 6B1, B2).

Third, we sought to verify whether FRAT1 or FRAT2 mediates the activation of the Wnt/β-catenin signalling pathway in GC cells with low miR-3648 expression. The forced expression of FRAT1 or FRAT2 resulted in the elevation of β‐catenin‐dependent luciferase activity, whereas miR-3648 suppressed it. In addition, co-expression of FRAT1, FRAT2, and miR-3648 reversed the effect of miR-3648 overexpression (Fig. 6C). Moreover, the increased activation of the Wnt/β-catenin signalling pathway of AGS and MKN45 cells transfected with the miR-3648 inhibitor was significantly rescued by FRAT1 or FRAT2 knockdown (Fig. 6D).

KYA1797K, a small molecule, inhibits the Wnt/β-catenin signalling pathway [41, 42]. We then determined whether KYA1797K suppresses β-catenin-dependent transcriptional activity in AGS and MKN45 cells via miR-3648. We showed that miR-3648 or KYA1797K attenuated luciferase activity compared to the m-NC + DMSO group. Moreover, both the miR-3648 and KYA1797K groups had lower levels of TOPFLASH transcription activity than the m-NC + DMSO or m-NC + KYA1797K groups (Fig. 6E).

To investigate the effect of miR-3648 or miR-3648 in combination with KYA1797K on cell migration and invasion, we performed transwell assays and scratch wound-healing in GC cells, respectively. The results showed that overexpression of miR-3648 or KYA1797K inhibited GC cell migration and invasion compared to those treated with m-NC + DMSO. Besides, the additive effect of miR-3648 and KYA1797K decreased the migration and invasion ability of GC cells compared to those treated with the m-NC + DMSO or m-NC + KYA1797K groups (Fig. 6F, G).

The FRAT1, FRAT2, GSK3b, β-catenin, TCF-4, MMP7, CD44, c-Myc, c-JUN, and HIF-1a proteins are key participants in the Wnt/β-catenin cell signalling pathway, in which aberrancies in their expression have been associated with malignant cell transformation [12, 14, 18, 35, 36, 38, 43, 44]. Therefore, the expression of these ten key proteins was measured via western blot assay in m-NC- and miR-3648 mimic-treated or miR-3648 inhibitor-treated cells. Representative blots for GC cells are shown in Fig. 6H1, H2. The relative expression of these proteins was further calculated via normalisation to GAPDH expression. The results showed that these ten proteins showed significantly abnormal expression patterns in miR-3648 mimic- or miR-3648 inhibitor-treated cells compared with the control cells (Fig. 6H1, H2).

These results demonstrate that miR-3648 inactivates the Wnt/β-catenin signalling pathway by targeting FRAT1 and FRAT2, affecting the migration and invasion of GC cells.

### miR-3648 is directly regulated by the transcription factor c-Myc

**Fig. 7:**
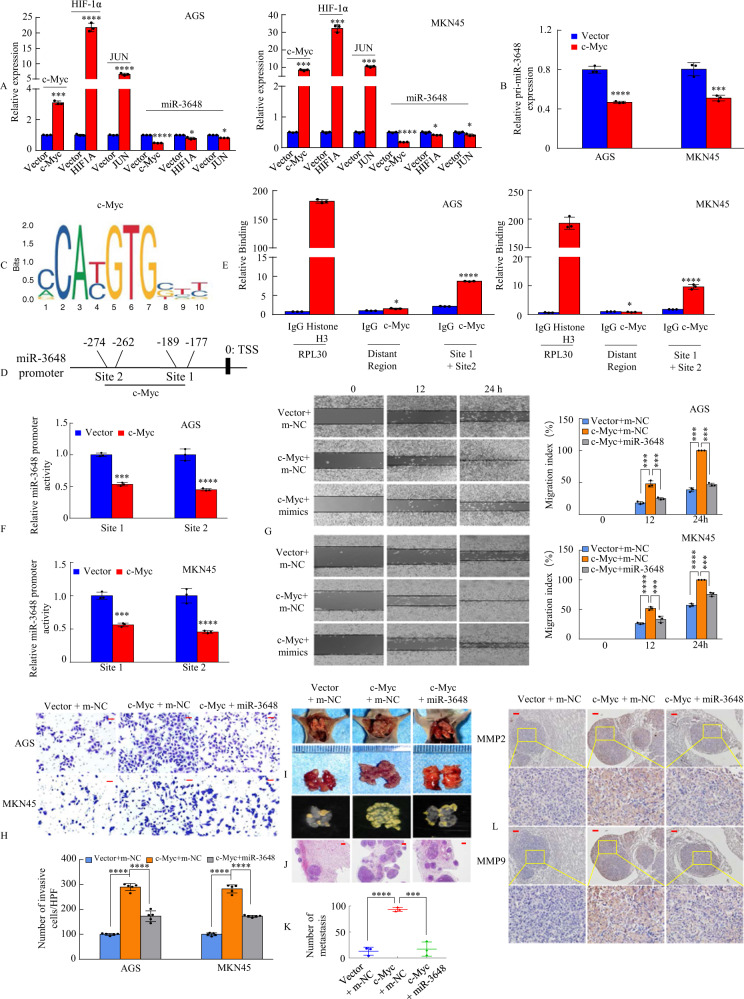
miR-3648 is regulated directly by the transcription factor c-Myc. **A**, **B** Overexpression of c-Myc, or HIF-1a, or JUN, or Vector was transfected into AGS and MKN45 cells. c-Myc, or HIF-1a, or JUN, or mature miR-3848 (**A**), or pri-miR-769 (**B**) relative expression levels were detected in AGS and MKN45 cell lines using qRT-PCR analysis. *P > 0.05; ***P < 0.01; ****P < 0.001. Two-tailed unpaired Student’s *t*-test. **C** The transcriptional factor c-Myc binding motif was predicted using Jaspar database. **D** The luciferase (Luc) reporter constructs contained the miR-3648 promoter with two potential c-Myc-binding sites. **E** ChIP-qPCR assay demonstrated the direct binding of c-Myc to the miR-3648 promoter in AGS and MKN45 cells. Gene enrichment was quantified relative to input controls by qPCR using primers specific for the promoter regions of miR-3648. PCR primers located in exon 3 of RPL30 and anti-Histone H3 antibody served as the positive control. Primers were used to amplify the served region containing the distant upstream miR-3648 promoter as the background. Results are shown as a fold change of qPCR value over IgG. Two-tailed unpaired Student’s *t*-test; *P > 0.05 and ****P < 0.001. **F** **c-**Myc transrepresses miR-3648 promoter activities in AGS and MKN45 cells. The miR-3648 promoter construct was cotransfected with c-Myc or vector, and the relative luciferase activity was determined. ***P < 0.01; ****P < 0.001. Two-tailed unpaired Student’s *t*-test. **G**, **H** The GC cell migration and invasion assays were performed. ***P < 0.01; ****P < 0.001. Two-tailed unpaired Student’s *t*-test. All experiments were repeated at least three times. **I** Mice were orthotopically transplanted with MKN45 cells (*n* = 3 in each group, one of three nude mice was shown. ***P < 0.01; ****P < 0.001. Two-tailed unpaired Student’s *t*-test. **J** The mice were killed and metastatic cancer tissues were stained with H&E. **K** The number of metastatic loci in the lungs was counted. ***P < 0.01; ****P < 0.001. **L** IHC staining of MMP2 and MMP9 expression. Scale bars, 50 μm in **H**, 200 μm in **J** and 100 μm in **L**.

The results mentioned above and previous reports have shown that a high number of target genes, such as c-Myc [13], MMP7 [45], cyclin D1 [16, 17], c-JUN [15, 46], and HIF-1α [18], in the Wnt/β-catenin signalling pathway have been identified. The ectopic expression of miRNAs is also regulated by transcription factor genes involved in the Wnt/β-catenin signalling pathway. Therefore, we investigated whether the transcription factors c-Myc, c-JUN, and HIF-1α can regulate miR-3648 expression levels in GC cells via a regulatory feedback loop. We showed that miR-3648 expression remained unchanged upon c-Jun and HIF-1a overexpression in GC cells. In contrast, ectopic expression of c-Myc led to a decreased expression of miR-3648 (Fig. 7A). Moreover, overexpression of c-Myc led to a decreased expression of the primary transcript of miR-3648 (pri-miR-3648) in GC cells (Fig. 7B). Next, we focused directly upstream within the 1-kb region of the transcription start site of miR-3648 and identified two putative c-Myc-binding sites (Fig. 7C) with at least 84% identity to the c-Myc consensus sequences (Fig. 7D), which were located at –189 to –177 bp (site 1) and –274 to –262 bp (site 2) in the miR-3648 promoter.

To determine whether c-Myc binds to the miR-3648 promoter region in vivo, we performed ChIP analysis in GC cells. The distance between the c-Myc binding sites 1 and 2 was 73 bp. The qPCR primer cannot be designed using online software (primer3 and primer3plus) because the GC content reached as high as 84.9% and it is difficult to separate sites close to each other. Thus, we combined c-Myc binding sites 1 and 2 to design primers for ChIP-qPCR. The results showed that the miR-3648 promoter region containing sites 1 and 2 exhibited significant enrichment after immunoprecipitation with an anti-c-Myc antibody (Fig. 7E). We then cloned the promoter regions of c-Myc site 1 or site 2 of miR-3648 upstream into a luciferase reporter plasmid. The AGS and MKN45 cells were transiently cotransfected with a c-Myc expression vector and a reporter plasmid. The dual-luciferase assay showed that the activity of miR-3648 sites 1 and 2 in c-Myc overexpressed cells decreased by approximately 2-fold compared to that in the vector-treated cells (Fig. 7F). These results suggest that c-Myc transrepresses the miR-3648 promoter in GC cells.

To determine whether miR-3648 affects cancer progression by regulating c-Myc expression in GC cells, we transfected c-Myc or vector, miR-3648 or m-NC, and c-Myc + miR-3648 into AGS and MKN45 cells. Compared with the control group, c-Myc overexpression enhanced the migration ability and invasiveness of GC cells in vitro, whereas the reintroduction of miR-3648 incompletely reversed the migration- and invasiveness-promoting effects of c-Myc (Fig. 7G, H).

To determine the effect of miR-3648 on c-Myc-mediated metastasis in vivo, MKN45/Vector + m-NC, MKN45/c-Myc + m-NC, and MKN45/c-Myc + miR-3648 cells were injected into the tail vein of nude mice to seed lung metastases (Fig. 7I and Supplementary Fig. 7). The presence of GC metastases in the lung was verified via H&E staining (Fig. 7J). The results showed that the number of metastatic nodules in the lung was significantly increased in mice injected with c-Myc cells compared to those injected with control cells. However, fewer lung metastatic nodules were observed in the c-Myc/miR-3648 group than in the c-Myc group (Fig. 7K). The expression of the cell metastasis markers MMP2 and MMP7 was confirmed via IHC (Fig. 7L).

These results suggest that c-Myc downregulates the expression level of miR-3648 in GC cells.

### miR-3648 expression levels are negatively correlated with c-Myc, FRAT1, and FRAT2 expression in human gastric samples

**Fig. 8:**
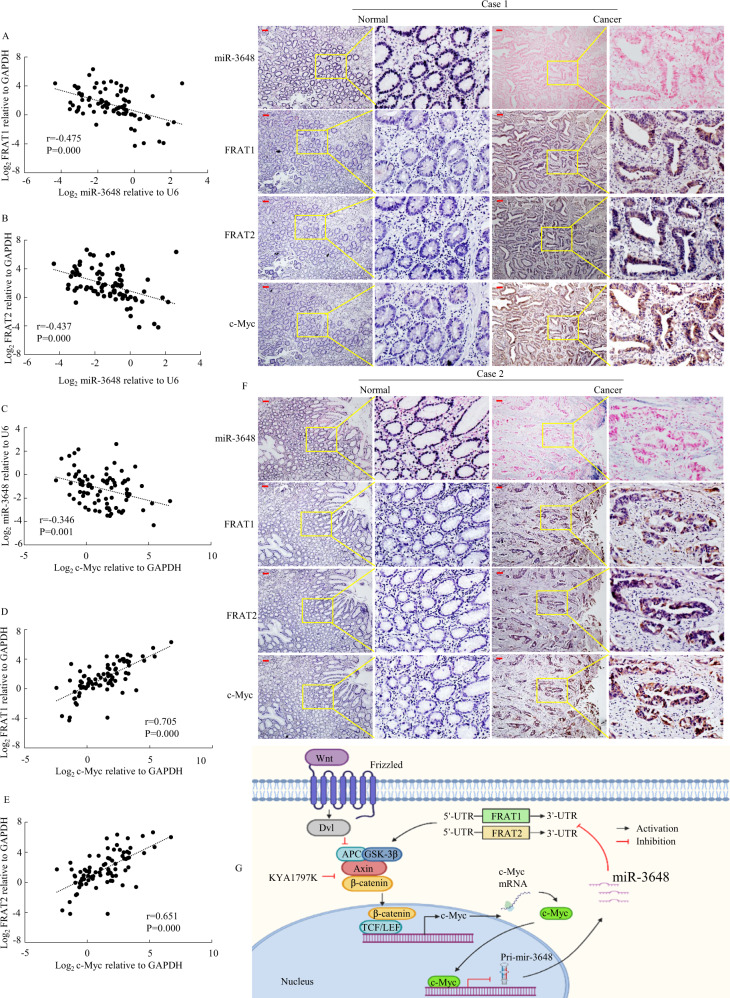
The correlation among expression of c-Myc, miR-3648, FRAT1 and FRAT2 in GC. **A**–**E** Spearman’s correlation analysis was used to determine the relationship between miR-3648 and FRAT1, that between miR-3648 and FRAT2, that between c-Myc and miR-3648, that between miR-3648 and FRAT1, and that between c-Myc and FRAT2 expression in 82 GC tissues using qPCR assay. **F** miR-3648, FRAT1, FRAT1 and c-Myc expression levels were detected by ISH and IHC assay. These figures were the representatives of gastric tissues from twelve cancerous and noncancerous patients. Scale bars, 50 μm. **G** A hypothetical model depicting roles of miR-3648, FRAT1, FRAT2 and c-Myc in GC cells.

To demonstrate the relationship between c-Myc, miR-3648, FRAT1, and FRAT2 expression levels in GC tissues, we detected the expression of c-Myc, miR-3648, FRAT1, and FRAT2 in 82 human GC tissues and paired normal tissues via qPCR. Spearman’s correlation analysis showed a negative correlation between miR-3648 and FRAT1 (Fig. 8A), between miR-3648 and FRAT2 (Fig. 8B) and between c-Myc and miR-3648 (Fig. 8C), and a positive correlation between c-Myc and FRAT1 (Fig. 8D) and between c-Myc and FRAT2 (Fig. 8E).

To test whether the entire miR-3648/FRAT1-FRAT2/c-Myc signalling axis was involved in GC, we evaluated the relationship between the expression levels of the molecules involved in these pathways and their association with the clinicopathological features of patients with GC. We proved that there was a correlation between the clinicopathological parameters of GC and miR-3648 expression (Supplementary Table 4). We found that the expression levels of FRAT1, FRAT2 and c-Myc (Supplementary Tables 5–7) were significantly correlated with GC lymph node metastasis and TNM stage.

Furthermore, we detected the expression of the four molecules using IHC and ISH assays. The expression levels of FRAT1, FRAT2 and c-Myc were significantly higher, whereas that of miR-3648 was significantly lower in GC tissues than in paired normal tissues of 12 gastric mucosa samples (Fig. 8F).

These data indicate that FRAT1, FRAT1, or c-Myc expression is negatively correlated with miR-3648 expression levels in patients with GC.

## Discussion

miRNAs play crucial roles in carcinogenesis and may regulate multiple gene targets to modulate metastatic progression in various cancer types [3, 4, 6, 15, 19]. However, the biological functions and mechanisms underlying the mode of action of miR-3648 in GC development and invasion have not yet been reported. In this study, we identified the function of miR-3648 in the downregulation of GC tumour growth and metastasis. miR-3648 targets FRAT1 and FRAT2, which are known to play an important role in the Wnt signalling pathway in GC progression. Moreover, we discovered that the miR-3648 promoter was negatively regulated by the Wnt pathway target gene c-Myc. Thus, these results suggest that the miR-3648/FRAT1-FRAT2/c-Myc negative feedback loop inhibits GC cell invasion and migration.

It is now well established that miRNAs are correlated with tumour proliferation and aggressiveness in gastrointestinal cancers. For example, miR-500a-5p directly targets HDAC2, which mediates the inhibition of CRC cell proliferation [47]. In this study, we first verified that miR-3648 was significantly downregulated in GC and that its expression was associated with a better prognosis in patients with GC. Overexpression of miR-3648 induces cell cycle arrest and inhibits the proliferation of GC cells. Moreover, ectopic expression of miR-3648 suppressed GC cell invasion and migration, which was consistent with previous reports that miR-188-5p inhibited the inhibition of growth, motility and invasion of GC cells [48]. Specifically, inhibition of miR-3648 facilitated GC development and progression, suggesting that miR-3648 can be used as a cancer biomarker.

The FRAT1 and FRAT2 genes are clustered in the human chromosome locus 10q24.1, and both genes have been previously identified as proto-oncogenes in a variety of tumours [11, 30, 36], including basal-like breast cancer [49], ovarian cancer [37], and GC [30]. However, whether miR-3648 participates in the regulation of FRAT1 or FRAT2 expression in GC remains unknown. In the current study, we identified FRAT1 and FRAT2 as major downstream effectors of miR-3648 from combined analyses using miRNA-seq and computational target prediction. We observed that miR-3648 negatively regulated the protein levels of FRAT1 or FRAT2 by targeting a specific binding site in the FRAT1 or FRAT2 3′-UTR sequence. Meanwhile, the overexpression of miR-3648 in FRAT1 or FRAT2-overexpressing GC cells regulated cell proliferation and metastasis both in vitro and in vivo. Thus, our study suggests that miR-3648 is a negative regulator of cell proliferation and metastasis by targeting FRAT1 and FRAT2 in GC.

The FRAT1 gene encodes a 279*-*amino-acids protein, whereas the FRAT2 gene encodes a 233-amino-acids protein, which is 77.3% identical to FRAT1 [38]. However, a correlation between FRAT1 and FRAT2 expression in GC has not been elucidated. Here, we revealed that FRAT1 physically binds to FRAT2 to promote metastasis in GC cells. First, we demonstrated that FRAT1 and FRAT2 may co-precipitate according to the GeneMANIA database. Second, we demonstrated a positive correlation between FRAT1 and FRAT2 expression using the GEPIA database and in 82 samples of GC tissues in our hospital. Third, co-immunoprecipitation and co-localisation experiments confirmed that FRAT1 interacts with FRAT2, which was consistent with the previous report by van der Wal et al. that TMEM98 binds to FRAT2 [50]. Fourth, FRAT1 synergises with FRAT2 to promote tumour metastasis in vitro and in vivo. Thus, we speculated that the FRAT1-FRAT2 axis might play a role in the progression of GC.

Wnt signalling is a highly conserved signalling pathway that plays a critical role in controlling embryonic and organ development, as well as cancer progression. For example, FZD7 is a positive regulator of the Wnt/β-catenin signalling pathway [51]. Its expression was related to the poor prognosis of GC patients. Axin is a negative regulator of the Wnt signalling cascade. Its expression is downregulated in GC tissues and associated with GC metastasis [52]. However, the role of FRAT1 and FRAT2 in modulating the Wnt signalling pathway in GC cells was not fully uncovered. In this study, we showed that ectopic expression of FRAT1 significantly enhanced β‐catenin‐dependent luciferase activity, whereas FRAT2 downregulation in FRAT1-overexpressing cells reversed the enhanced effect, which is consistent with TCTP and Wnt-5a heighten β-catenin/TCF-4 transcription activity using a TOPflash/FOPflash report gene assay [53, 54]. Therefore, FRAT1 or FRAT2 alone and FRAT1 synergises with FRAT2 to activate Wnt/β-catenin signalling in GC cells.

Subsequently, we investigated whether miR-3648 can inhibit GC tumour metastasis by modulating the FRAT1-FRAT2/Wnt/β-catenin signalling pathway in GC. Results of the TOPFLASH/FOPFLASH reporter assays showed that FRAT1 or FRAT2 increased the Wnt/β-catenin activity, whereas miR-3648 decreased the Wnt/β-catenin activity in GC cells. Moreover, co-transfection of FRAT1 or FRAT2 and miR-3648 partially reversed the effect of miR-3648 suppression on Wnt/β-catenin activity. Western blot analysis showed that miR-3648 suppressed Wnt*/*β-catenin target gene expression. Thus, miR-3648 inactivates the Wnt/β-catenin signalling pathway by targeting FRAT1 and FRAT2.

Recent reports have revealed that miRNAs in combination with Wnt/β-catenin signalling antagonists negatively regulate the Wnt/β-catenin signalling pathway in tumour metastasis [19, 55]. Consistently, we demonstrated that the miR-3648 and/or Wnt/β-catenin inhibitor-KYA1797K-treated cells showed lower levels of luciferase activity than the control. In addition, miR-3648 synergises with KYA1797K and inhibits GC cell migration and invasion ability compared to the control group. Thus, both miR-3648 and KYA1797K are potential therapeutic strategies for GC patients.

An increasing amount of evidence shows that the deregulated expression of the c-Myc proto-oncogene by aberrant Wnt/β-catenin signalling drives tumour cell migration and invasion [13, 43, 56]. As a well-studied transcription factor, c-Myc can regulate the transcription of several miRNAs by binding to their promoter regions, thereby modulating cancer progression. For instance, c-Myc can inhibit the expression of miR-200b via binding to the promoter region of the miR-200b gene [57]. The c-Myc-miR-200b-PRDX2 axis regulates CRC progression [58]. Here, miR-3648 is a direct transcriptional target of c-Myc, as demonstrated by c-Myc directly binding to and transrepressing the miR-3648 promoter. This result provides a mechanism by which c-Myc inhibits the expression of miR-3648, regulating GC metastasis and invasion. Furthermore, the above-mentioned results showed that miR-3648 targets FRAT1 and FRAT2, and that FRAT1/FRAT2 positively regulates the Wnt signalling pathway and downregulates c-Myc expression. These findings suggest that miR-3648/FRAT1-FRAT2/c-Myc forms a negative feedback loop, which could inhibit GC cell invasion and metastasis.

In conclusion, as illustrated in our working model in Fig. 8G, we have shown that miR-3648 inactivates Wnt/β-catenin signalling by directly targeting their regulators, FRAT1 and FRAT2. In turn, inactivation of Wnt/β-catenin signalling leads to the upregulation of miR-3648 expression by reducing the binding of c-Myc to the miR-3648 promoter region. Therefore, miR-3648/FRAT1-FRAT2/c-Myc forms a negative feedback loop, which regulates GC cell invasion and metastasis. Our results showed that miR-3648 may be a novel prognostic marker and a treatment target for GC.

## Materials and methods

Available in Supplementary information.

## Supplementary information

Supplementary information

## Data Availability

The datasets used and analysed during the current study are available from the corresponding author upon reasonable request.
