# Supplementary material for: The miR-3648/FRAT1-FRAT2/c-Myc negative feedback loop modulates the metastasis and invasion of gastric cancer cells
Source: Oncogene. 2022 Sep 24;41(43):4823–38. doi: 10.1038/s41388-022-02451-2 (PMC9586869; doi:10.1038/s41388-022-02451-2)
Supplement: Supplementary file 1 — Supplementary information [file 41388_2022_2451_MOESM1_ESM.docx]

**Materials and Methods**

**Patients and specimens**

This study included 82 patients with GC. Fresh GC tissues and adjacent non-cancerous tissues were collected from patients pathologically validated to have GC who underwent gastrectomy at Nanfang Hospital, Southern Medical University from 2017 to 2019. None of the patients received chemotherapy or radiation therapy before gastrectomy. All GC cases were confirmed by two senior pathologists and were staged according to the American Joint Committee on Cancer (AJCC) TNM classification of gastric cancer (7th ed., 2010). This study was approved by the Ethics Committee of Nanfang Hospital. Informed consent of the patient has been obtained.

**Reagents, cell lines and cell culture**

KYA1797K was dissolved in dimethyl sulfoxide (DMSO; Sigma-Aldrich) for the in vitro studies. The non-tumorigenic immortalised human normal gastric mucosal cell line GES-1 and GC cell lines (HGC-27, MKN45, MGC-803, BGC-823, SGC-7901, AGS, and MKN74) were purchased from the Type Culture Collection of the Chinese Academy of Sciences (Shanghai, China). Cells were cultured in DMEM (Gibco, Carlsbad, CA, USA) medium containing 10% foetal bovine serum and 1% penicillin/streptomycin in a humidified incubator at 37 °C with an atmosphere of 5% CO_2_.

**RNA extraction and qRT-PCR**

Total RNA was extracted from cells or tissues using RNAiso Plus (9109, Takara Biomedical Technology, Dalian, China). Specific PCR primers for miR-3648 (#HmiRQP1926) and snRNA U6 (#HmiRQP9001) were purchased from Gene Copoeia (San Diego, CA, USA). The All-in-One^TM^ miRNA First-Strand cDNA Synthesis Kit (Gene Copoeia) and All-in-One™ miRNA qRT-PCR Detection Kit (Gene Copoeia) for qPCR detection were used according to the manufacturer’s instructions. For mRNA expression analysis, qPCR was performed using the PrimeScript RT reagent kit (RR036A, Takara) and SYBR Premix Ex Taq (RR420B, Takara). SnRNA U6 was used as an internal control for detecting miRNA expression, and GAPDH was used as an internal control to detect mRNA expression. The 2^-ΔΔCt^ method was used for data analysis. The primers used are listed in Supplementary Table 1.

***In situ* hybridisation (ISH)**

ISH was performed on paraffin-embedded tissue sections, and the digoxigenin-labelled oligonucleotide probe was purchased from Boster (Wuhan, China). The probe sequence was as follows: 5ʹ-CCCTCGGCGATCCCCGCGGCT-3ʹ. The experiment was performed according to the usage guidelines of the Boster ISH detection kit.

**Lentiviral infection, oligonucleotide and plasmid transfections**

miR-3648 m-NC (sense, 5ʹ-UUCUCCGAACGUGUCACGUTT-3ʹ), miR-3648 mimic (sense, 5ʹ-AGCCGCGGGGAUCGCCGAGGG-3ʹ), i-NC (sense, 5ʹ-CAGUACUUUUGUGUAGUACAA-3ʹ), and miR-3648 inhibitor (sense, 5ʹ- CCCUCGGCGAUCCCCGCGGCU-3ʹ) were synthesised by Genepharma (Shanghai, China). FRAT1, c-Myc, JUN, and HIF-1a plasmids were purchased from Vigene Biosciences (Shandong, China), and pEnter plasmid was used as a control. The FRAT2 plasmid was constructed by Kidan Bio Co. Ltd (Guangzhou, China), and the pcDNA3.1(+) vector plasmid was used as a control. The FRAT1 siRNA sequences were as follows: FRAT1 siRNA-1-sense, 5ʹ- GCAGTTACGTGCAAAGCTT-3′; FRAT1 siRNA-2-sense, 5ʹ- GGCTTCATTCGCGACGGCT′-3; and FRAT1 siRNA-3-sense, 5ʹ- GAGCTGGCAAGCAGGGCAT-3′. The FRAT2 siRNA sequences were as follows: FRAT2 siRNA-1-sense, 5ʹ-GCUACAAUUGCUGUGCAAATT-3′; FRAT2 siRNA-2-sense, 5ʹ-GGGAAACCUCAUCAAGGAATT′-3; and FRAT2 siRNA-3-sense, 5ʹ- GCAUCGACUUGUACCUCACUUTT-3′. Transfection was conducted using Lipofectamine 3000 (Invitrogen, Foster City, CA, USA) following the manufacturer’s instructions.

Overexpression of miR-3648, FRAT1, FRAT2, and c-Myc and knockdown of FRAT2 were accomplished using a lentivirus packaging system. All lentiviruses were purchased from GeneChem Co., Ltd. (Shanghai, China). For miR-3648 overexpression, we used a lentiviral expression vector (Ubi-MCS-SV40-Cherry) containing the red fluorescent protein gene. For FRAT1, FRAT2, and c-Myc overexpression, we used a lentiviral expression vector (Ubi-MCS-3FLAG-SV40-EGFP-IRES-Hygromycin) containing the green fluorescent protein gene. Knockdown of FRAT2 was mediated by the designed shRNAs. The FRAT2 sense shRNA (NM_012083) sequence (FRAT2 siRNA-2) was 5′- CCGGGGGAAACCUCAUCAAGGAATCAAGAGTTCCTTGATGAGGTTTCCCTTTTTG-3′ and the sense scramble sequence was 5′- CCGGTTCTCCGAACGTGTCACGTCTCGAGACGTGACACGTTCGGAGAATTTTTG-3′. The AGS and MKN45 cell lines were transfected with lentiviral particles according to the manufacturer’s instructions and selected using puromycin or hygromycin. The transfection effect was verified via fluorescence intensity analysis, qPCR, and western blotting.

**Flow cytometry**

Six-well plates were used to incubate and transfect cells. After 48 h, the cells were collected and stained with the Beyotime Cell Cycle and Apoptosis Analysis Kit (Shanghai, China). Briefly, the cells were fixed with 70% ethanol overnight and stained with propidium iodide. A flow cytometer was used to detect red fluorescence at an excitation wavelength of 488 nm. FlowJo was used for the cell DNA content and light scattering analyses.

**Western blot**

Proteins were extracted using a lysis buffer. Protein lysates were separated using 10% SDS-PAGE and transferred to Immobilon-P membranes (Millipore, Billerica, MA, USA). The membrane was blocked with 5% non-fat milk for 1 h at room temperature and incubated with the primary antibodies overnight at 4 °C. After washing, the membrane was incubated with the corresponding horseradish peroxidase-conjugated secondary antibodies (1:6000; ZB2301 and ZB-2305, ZSGB-bio, Beijing, China) for 1 h at room temperature. Proteins were visualised using an Immobilon ECL kit (Millipore). The primary antibodies used in this study include: CDK4 (D9G3E) (1:1000; #12790; CST), CDK6 (D4S8S) (1:1000; #13331; CST), Cyclin B1 (D5C10) (1:1000; #12231; CST), Cyclin D1 (E3P5S) (1:1000; #55506; CST), FRAT1 (1:1000; ab137391; Abcam), FRAT2 (1:1000; orb29820; Biorbyt), GSK3B (1:1000; 22104-1-AP; Proteintech), β-catenin (D10A8) (1:1000; #8480; CST), TCF4 (1:500; 13838-1-AP; Proteintech), c-Myc (1:5000; 67447-1-Ig; Proteintech), MMP7 (1:500;10374-2-AP; Proteintech), CD44 (1:2000; 15675-1-AP; Proteintech), JUN (1:500; 22114-1-AP; Proteintech), HIF1A (1:2000; 66730-1-Ig; Proteintech), and GAPDH (MC4) (1:5000; RM2002; Beijing Ray Antibody Biotech).

**Cell proliferation assays**

Transfected cells were seeded in 96-well plates at a density of 3000 cells per well for routine culture. To determine cell proliferation, Cell Counting Kit-8 (CCK-8) (Dojindo, Japan) was added to the cells and were incubated at 37 °C for 1 h. The density of cells was measured using an automatic plate reader (Beckman, California, USA) at a wavelength of 450 nm at the corresponding time points.

**Colony formation assay**

The cells were digested with 0.25% trypsin and pipetted into a single tube. The cells were seeded at a density of 200 cells per well in a 12-well plate and placed in a humidified incubator at 37 °C with an atmosphere of 5% CO_2_ for 2 weeks. The supernatant was then discarded, and the plate was washed with PBS. The cells were then fixed with methanol and stained with 0.05% crystal violet. The number of colonies was calculated.

**EdU incorporation assay**

The isolated GC cells from each group were inoculated into 96-well plates in triplicate at a density of 1×10^4^ cells per well and incubated overnight. Next, 100 μL of 50 μM EdU medium was added to each well and incubated for 2 h. Cell-Light EdU Apollo567 In Vitro Kit (Ribobio, Guangzhou, China) was used to detect cell proliferation following the manufacturer’s instructions. An inverted fluorescence microscope (Olympus, Tokyo, Japan) was used for observing the cells. Three random fields were selected to image each well. ImageJ software was used to analyse the percentage of proliferating cells in each field.

**Cell wound healing and cell invasion assay**

For migration assay, transfected cells were inoculated into six-well plates at a density of 1×10^6^ cells per well and cultured to confluence. A 10 μL pipette tip was used to scratch the centre of the plate and the cells that disintegrated from the wound were carefully rinsed away. Mitomycin C was used to inhibit cell proliferation. Images were taken at the time of settling. The migration index was calculated as follows: migration index = [(initial wound width-wound width at the settlement time)/initial wound width]×100%.

For the cell invasion assay, Matrigel (BD Biosciences, Boston, Massachusetts, USA) was used to coat the upper layer of a transwell chamber (Corning, USA). Next, 500 μL of a culture medium containing 20% FBS was added to each well of a 24-well plate. Transfected cells at a density of 1×10^5^ were added to the upper chamber and cultured for 48 h. Afterwards, the transwell chamber was removed, and the cells adhered to the upper surface. The cells that adhered to the lower surface of the membrane were visualised with 0.05% crystal violet. At least five random fields were counted under an upright microscope (Olympus, Tokyo, Japan).

**RNA sequencing**

The AGS cells were transfected with m-NC or miR-3648 mimics. After 48 h, total RNA was extracted from the cells and RNA sequencing was performed (Aksomics, Shanghai, China). Briefly, the total RNA samples were subjected to agarose electrophoresis, and Nanodrop was used for quality inspection and quantification. Afterwards, the mRNA was enriched with oligo(dT) magnetic beads. Library construction was performed using a KAPA Stranded RNA-Seq Library Prep Kit (Illumina, San Diego, CA, USA). After RNA fragmentation, random primers were used to invert the first strand of the cDNA. dUTP was then added to synthesise the second strand of cDNA. After repairing the double-stranded cDNA end and adding dA-tailing, Illumina matching adapters were connected to the final library via PCR amplification. The inspection quality of the constructed library was determined using Agilent 2100 (Santa Clara, CA, USA). Sequencing was performed using an Illumina NovaSeq 6000 sequencer. Solexa pipeline version 1.8 (Off-Line Base Caller software, version 1.8) software for image processing and base identification. The R software package Ballgown was used to calculate the FPKM at the gene and transcript levels. The RNA sequencing data was deposited in GEO with Series record GSE204669.

***In vivo*** **metastasis assay**

All BALB/c nude mice were purchased from the Central Laboratory of Animal Science at Southern Medical University and maintained in a pathogen-free environment. The study was approved by the Southern Medical University Experimental Animal Ethics Committee. In the tail vein metastasis assay, a total of 5×10^6^ lentivirus-transfected cells were injected into the tail vein of nude mice (female, 4–6 weeks old, randomly assigned to different groups and each group have at least three mice). After 30 days, the mice were sacrificed. The lung tissues were dissected and tested for bioluminescence. Metastatic tumours were detected via haematoxylin and eosin staining and quantified by calculating the number of metastases in each section.

**Haematoxylin and Eosin (H&E) staining**

Formalin-fixed and paraffin-embedded sections were stained with haematoxylin and eosin. The sections were deparaffinised in xylene and hydrated with a series of decreasing concentrations of ethanol (100%, 95%, 85%, and 75%). The sections were stained with haematoxylin for 5 min, differentiated with 1% alcohol hydrochloric acid for 2 s, and stained with eosin for 1 min.

**Immunohistochemistry**

Paraffin-embedded tissues were prepared and cut into 4-μm-thick sections. The sections were then transferred to glass slides and deparaffinised with xylene. After hydration with decreasing concentrations of ethanol, 3% hydrogen peroxide was used to block endogenous peroxidase activity. Citrate buffer was used for antigen retrieval. The sections were then incubated with the primary antibody at 4 °C overnight. After washing, sections were incubated with HRP labelled goat anti-rabbit IgG or HRP labelled goat anti-mouse IgG (PV-6001 and PV-6002, ZSGB-bio, Beijing, China). Freshly prepared DAB complex (ZLI-9017, ZSGB-bio, Beijing, China) was used for staining. The following primary antibodies were used: FRAT1 (1:100; ab137391; Abcam), FRAT2 (1:30; orb29820; Biorbyt), c-Myc (1:1000; 67447-1-Ig; Proteintech), MMP2 (1:100; 10373-2-AP; Proteintech), and MMP9 (1:100; 10375-2-AP; Proteintech).

**Co-immunoprecipitation (Co-IP) assay**

Cells were transfected with plasmids encoding proteins tagged with FLAG or HA. The proteins were collected in lysis buffer and incubated with 2 μg of antibody. Protein A/G immunoprecipitation magnetic beads were used to enrich the antigen-antibody complexes. After thorough washing, western blotting was performed to detect the potential interacting proteins. The specific antibodies used were as follows: HA Tag (51064-2-AP; Proteintech) and Flag Tag (D6W5B, #14793; CST).

**Immunofluorescence (IF) assay**

The transfected cells were inoculated on the processed coverslips and cultivated to a confluency of 30%–40%. The cells were fixed with 4% paraformaldehyde for 30 min at room temperature. The cells were then permeabilised with 0.2% Triton X-100 for 5 min and blocked with 1% BSA. The cells were then incubated with primary antibodies against FRAT1 (1:100, ab137391; Abcam) and HA (1:100, RM1004; Beijing Ray Antibody Biotech). Thereafter, the cells were incubated with goat anti-mouse IgG conjugated with Alexa Fluor 594 (red, ZF-0513, ZSGB-bio, Beijing, China) and goat anti-rabbit IgG conjugated with Alexa Fluor 488 (green, ZF-0511, ZSGB-bio, Beijing, China) at a dilution of 1:100. Cell nuclei were stained using Hoechst 33258 and inspected using a fluorescence microscope (Olympus, Japan) at 400× magnification.

**Chromatin immunoprecipitation (Chip)**

SimpleChIP Enzymatic Chromatin IP Kit (Magnetic Beads, #9003, CST) was used to perform chromatin immunoprecipitation according to the manufacturer’s instructions. Briefly, cells grown in a 10-cm culture dish were treated with 1% formaldehyde to cross-link chromatin-related proteins with DNA. Micrococcal nuclease and sonication were applied to the cell lysate to cut DNA into fragments of 150–900 bp. The lysate was incubated with antibodies specific to c-Myc (67447-1-Ig; Proteintech) or with normal rabbit IgG. Antibodies against Histone H3 (D2B12) (#4620) and human RPL30 exon 3 primers (#7014) were used as positive controls. The chromatin supernatants were incubated with 30 μL protein A/G magnetic beads at 4 °C on a rotator overnight. The protein-DNA complexes were eluted and purified to obtain pure DNA. Amplification of the binding sites was verified using qRT-PCR. The primers used are listed in Supplementary Table 1.

**Luciferase assay**

The reporter constructs pTOPFLASH and pFOPFLASH, in which the luciferase gene was driven by the wild-type or dominant negative mutant consensus 3× T cell factor/lymphoid enhancer factor binding element (TBE), respectively, were kindly provided by Professor Schneikert J. of the University Erlangen-Nürnberg, Germany. We then constructed a series of plasmids containing *the* *promoter* *fragment*s of miR-3648 into the pGL3-Basic vector. The primers used are listed in Supplementary Table 1. FastDigest restriction enzymes *Nhe*I and *Kpn*I (Thermo Fisher, Waltham, MA, USA) were used according to the manufacturer’s instructions. For the luciferase assay, cells were transiently transfected with Lipofectamine 3000. As previously mentioned, the Dual-Glo Luciferase Assay System (Promega, Madison, WI, USA) was used to sequentially measure the firefly and Renilla luciferase activities from a single sample.

**Luciferase activity assay for the 3ʹ-UTR study**

The luciferase reporter plasmid carrying the WT or mutated (MUT) FRAT1/FRAT2 3ʹ-UTR (pMIR-report-FRAT1-WT-3ʹ-UTR and pMIR-report-FRAT1-MUT-3ʹ-UTR, pMIR-report-FRAT2-WT-3ʹ-UTR, and pMIR-report-FRAT2-MUT-3ʹ-UTR, respectively) were transfected into GC cells along with the miR-3648 mimics using Lipofectamine 3000 (Invitrogen). After transfection (36–48 h), the cells were lysed, and luciferase activity was measured using the Dual-Luciferase Reporter Assay system (Promega, Madison, Wisconsin, USA). The sequences of FRAT1-WT-3ʹ-UTR, FRAT1-MUT-3ʹ-UTR, FRAT2-WT-3ʹ-UTR, and FRAT2-MUT-3ʹ-UTR are shown in Fig. 3E.

**Statistical analysis**

Statistical analysis was performed using the SPSS statistical software package (standard version 22.0, Chicago, Illinois). Quantitative data obtained from biological replicate experiments are shown as means ± standard deviations. All experiments were conducted with at least three independent replicates. The survival rates were calculated using the Kaplan-Meier curves, and the log-rank test was used to test the differences in survival rates between the two groups. The significance of the differences between groups was analysed using Student’s t-test. The F-test was performed before the groups were statistically compared. One-way analysis of variance was used to compare multiple groups. Statistical significance was set at P < 0.05.

Supplementary table 1. Primers Used in This Study

| Name | Orientation | Sequence |
| --- | --- | --- |
| GAPDH | Forward | AAATCCCATCACCATCTTCC |
|  | Reverse | TCACACCCATGACGAACA |
| c-Myc | Forward | TCTGGATCACCTTCTGCTGG |
|  | Reverse | TGTTGCTGATCTGTCTCAGG |
| JUN | Forward | TCCAAGTGCCGAAAAAGGAAG |
|  | Reverse | CGAGTTCTGAGCTTTCAAGGT |
| HIF1A | Forward | CACCACAGGACAGTACAGGAT |
|  | Reverse | CGTGCTGAATAATACCACTCACA |
| Pri-miR-3648 | Forward | GTGGTCTCTCGTCTTCTC |
|  | Reverse | ACGGACGCCTCGGGGAAG |
| ChIP-site 1 plus site 2 | Forward | TCACGTCCGTTGGTGGCC |
|  | Reverse | CCTCCGGGAAGCCCACC |
| ChIP-Distant region | Forward | TCCACGGCTAGAGTGCAATG |
|  | Reverse | CCTCCCTGTAATCCCCGCTA |
| Luc-Site 1 | Forward | cggggtaccCCCCGCGTGGGGCCCGGT |
|  | Reverse | ctagctagcCGACCCGGTTCGGAAGAG |
| Luc-Site 2 | Forward | cggggtaccCGCCTCGTGGGGCGCCGC |
|  | Reverse | ctagctagcCGACCCGGTTCGGAAGAG |

Supplementary table 2. miRNA signature predicts survival

in gastric cancer (p < 0.01, upregulated in deceased)

| miRNA | Log Rank（p value） |
| --- | --- |
| hsa-miR-653-5p | 0.000282 |
| hsa-miR-143-5p | 0.000906 |
| hsa-miR-409-5p | 0.00116 |
| hsa-miR-100-5p | 0.00156 |
| hsa-miR-3145-3p | 0.00273 |
| hsa-miR-136-5p | 0.00326 |
| hsa-miR-99b-5p | 0.0042 |
| hsa-miR-328-3p | 0.00445 |
| hsa-miR-145-3p | 0.005 |
| hsa-miR-145-5p | 0.00757 |
| hsa-miR-139-5p | 0.00791 |
| hsa-miR-302a-5p | 0.00797 |
| hsa-miR-199a-5p | 0.00819 |
| hsa-miR-1228-3p | 0.00889 |
| hsa-miR-125a-5p | 0.0089 |

Supplementary table 3. miRNA signature predicts survival

in gastric cancer (p < 0.01, upregulated in living )

| miRNA | Log Rank（p value） |
| --- | --- |
| hsa-miR-1306-3p | 0.00254 |
| hsa-miR-3648 | 0.00735 |
| hsa-miR-2115-3p | 0.00914 |
| hsa-let-7f-5p | 0.0013 |
| hsa-miR-7-5p | 0.00601 |
| hsa-miR-96-3p | 0.00653 |
| hsa-miR-942-5p | 0.00662 |
| hsa-miR-135b-3p | 0.00731 |
| hsa-miR-423-5p | 0.00819 |
| hsa-miR-221-3p | 0.00848 |
| hsa-miR-15b-5p | 0.00969 |

Supplementary table 4. Correlation between miR-3648 expression level and the clinicopathological parameters of gastrc carcinoma

| **Characteristitis** | **Case** | **miR-3648 expression (％)** | | **χ2 value** | **P value** |
| --- | --- | --- | --- | --- | --- |
|  |  | **Low** | **High** |  |  |
| Sex |  |  |  |  |  |
| Male | 60 | 30(50.0%) | 30(50.0%) | 0.000 | 1 |
| Female | 22 | 11(50.0%) | 11(50.0%) |  |  |
| Age(yr) |  |  |  |  |  |
| < 60 | 34 | 17(50.0%) | 17(50.0%) | 0.000 | 1 |
| ≥ 60 | 48 | 24(50.0%) | 24(50.0%) |  |  |
| Tumor size |  |  |  |  |  |
| < 3cm | 13 | 5(38.5%) | 8(61.5%) | 0.823 | 0.364 |
| ≥ 3cm | 69 | 36(52.2%) | 33(47.8%) |  |  |
| Differentiantion |  |  |  |  |  |
| Well | 9 | 3(33.3%) | 6(66.7%) | 6.136 | 0.047* |
| Moderate | 24 | 8(33.3%) | 16(66.7%) |  |  |
| Poor | 49 | 30(61.2%) | 19(38.8%) |  |  |
| Depth of invasion |  |  |  |  |  |
| T1 - T2 | 18 | 1(5.56%) | 17(94.4%) | 18.222 | < 0.001* |
| T3 - T4 | 64 | 40(62.5%) | 24(37.5%) |  |  |
| Lymph node metastasis |  |  |  |  |  |
| Present | 63 | 38(60.3%) | 25(39.7%) | 11.577 | 0.001* |
| Absent | 19 | 3(15.8%) | 16(84.2%) |  |  |
| TNM stage(AJCC) |  |  |  |  |  |
| I - II | 25 | 5(20.0%) | 20(80.0%) | 12.947 | < 0.001* |
| III - IV | 57 | 36(63.2%) | 21(36.8%) |  |  |

Supplementary table 5. Correlation between FRAT1 expression and the clinicopathological parameters of gastrc carcinoma

| Characteristitis | Case | FRAT1 expression (%) | | χ2 value | P value |
| --- | --- | --- | --- | --- | --- |
|  |  | Low | High |  |  |
| Sex |  |  |  |  |  |
| Male | 60 | 27(45.0%) | 33(55.0%) | 2.236 | 0.135 |
| Female | 22 | 14(63.6%) | 8(36.4%) |  |  |
| Age(yr) |  |  |  |  |  |
| < 60 | 34 | 21(61.8%) | 13(38.2%) | 3.216 | 0.073 |
| ≥ 60 | 48 | 20(41.7%) | 28(58.3%) |  |  |
| Tumor size |  |  |  |  |  |
| < 3cm | 13 | 8(61.5%) | 5(38.5%) | 0.823 | 0.364 |
| ≥ 3cm | 69 | 33(47.8%) | 36(52.2%) |  |  |
| Differentiantion |  |  |  |  |  |
| Well | 9 | 3(33.3%) | 6(66.7%) | 1.697 | 0.428 |
| Moderate | 24 | 11(45.8%) | 13(54.2%) |  |  |
| Poor | 49 | 27(55.1%) | 22(44.9%) |  |  |
| Depth of invasion |  |  |  |  |  |
| T1-T2 | 18 | 14(77.8%) | 4(22.2%) | 7.118 | 0.008* |
| T3-T4 | 64 | 27(42.2%) | 37(57.8%) |  |  |
| Lymph node metastasis |  |  |  |  |  |
| Present | 63 | 25(39.7%) | 38(60.3%) | 11.577 | 0.001* |
| Absent | 19 | 16(89.5%) | 3(10.5%) |  |  |
| TNM stage(AJCC) |  |  |  |  |  |
| I - II | 25 | 19(76.0%) | 6(24.0%) | 9.725 | 0.002* |
| III - IV | 57 | 22(38.6%) | 35(61.4%) |  |  |

Supplementary table 6. Correlation between FRAT2 expression and the clinicopathological parameters of gastrc carcinoma

| Characteristitis | Case | FRAT2 expression (％) | | χ2 value | P value |
| --- | --- | --- | --- | --- | --- |
|  |  | Low | High |  |  |
| Sex |  |  |  |  |  |
| Male | 60 | 29(48.3%) | 31(51.7%) | 0.248 | 0.618 |
| Female | 22 | 12(54.5%) | 10(45.5%) |  |  |
| Age(yr) |  |  |  |  |  |
| < 60 | 34 | 20(58.8%) | 14(41.2%) | 1.809 | 0.179 |
| ≥ 60 | 48 | 21(43.8%) | 27(56.2%) |  |  |
| Tumor size |  |  |  |  |  |
| < 3cm | 13 | 5(38.5%) | 8(61.5%) | 0.823 | 0.364 |
| ≥ 3cm | 69 | 36(52.2%) | 33(47.8%) |  |  |
| Differentiantion |  |  |  |  |  |
| Well | 9 | 4(44.4%) | 5(55.6%) | 1.288 | 0.525 |
| Moderate | 24 | 10(41.7%) | 14(58.3%) |  |  |
| Poor | 49 | 27(55.1%) | 22(44.9%) |  |  |
| Depth of invasion |  |  |  |  |  |
| T1 - T2 | 18 | 12(66.7%) | 6(33.3%) | 2.563 | 0.109 |
| T3 - T4 | 64 | 29(45.3%) | 35(54.7%) |  |  |
| Lymph node metastasis |  |  |  |  |  |
| Present | 63 | 26(41.3%) | 37(58.7%) | 13.562 | < 0.001* |
| Absent | 19 | 15(78.9%) | 4(21.1%) |  |  |
| TNM stage(AJCC) |  |  |  |  |  |
| I - Ⅱ | 25 | 17(68.0%) | 8(32.0%) | 4.661 | 0.031* |
| Ⅲ - Ⅳ | 57 | 24(42.1%) | 33(57.9%) |  |  |

Supplementary table 7. Correlation between c-Myc expression and the clinicopathological parameters of gastrc carcinoma

| **Characteristitis** | **Case** | **MYC expression (％)** | | **χ2 value** | **P value** |
| --- | --- | --- | --- | --- | --- |
|  |  | **Low** | **High** |  |  |
| Sex |  |  |  |  |  |
| Male | 60 | 28(46.7%) | 32(53.3%) | 0.994 | 0.319 |
| Female | 22 | 13(59.1%) | 9(40.9%) |  |  |
| Age(yr) |  |  |  |  |  |
| < 60 | 34 | 21(61.8%) | 13(38.2%) | 3.216 | 0.073 |
| ≥ 60 | 48 | 20(41.7%) | 28(58.3%) |  |  |
| Tumor size |  |  |  |  |  |
| < 3cm | 13 | 8(61.5%) | 5(38.5%) | 0.823 | 0.364 |
| ≥ 3cm | 69 | 33(47.8%) | 36(52.2%) |  |  |
| Differentiantion |  |  |  |  |  |
| Well | 9 | 4(44.4%) | 5(55.6%) | 1.292 | 0.524 |
| Moderate | 24 | 10(41.7%) | 14(58.3%) |  |  |
| Poor | 49 | 27(55.1%) | 22(44.9%) |  |  |
| Depth of invasion |  |  |  |  |  |
| T1 - T2 | 18 | 14(77.8%) | 4(22.2%) | 7.118 | 0.008* |
| T3 - T4 | 64 | 27(42.2%) | 37(57.8%) |  |  |
| Lymph node metastasis |  |  |  |  |  |
| Present | 63 | 26(41.3%) | 37(58.7%) | 8.289 | 0.004* |
| Absent | 19 | 15(78.9%) | 4(21.1%) |  |  |
| TNM stage(AJCC) |  |  |  |  |  |
| I - Ⅱ | 25 | 17(68.0%) | 8(32.0%) | 4.661 | 0.031* |
| Ⅲ - Ⅳ | 57 | 24(42.1%) | 33(57.9%) |  |  |


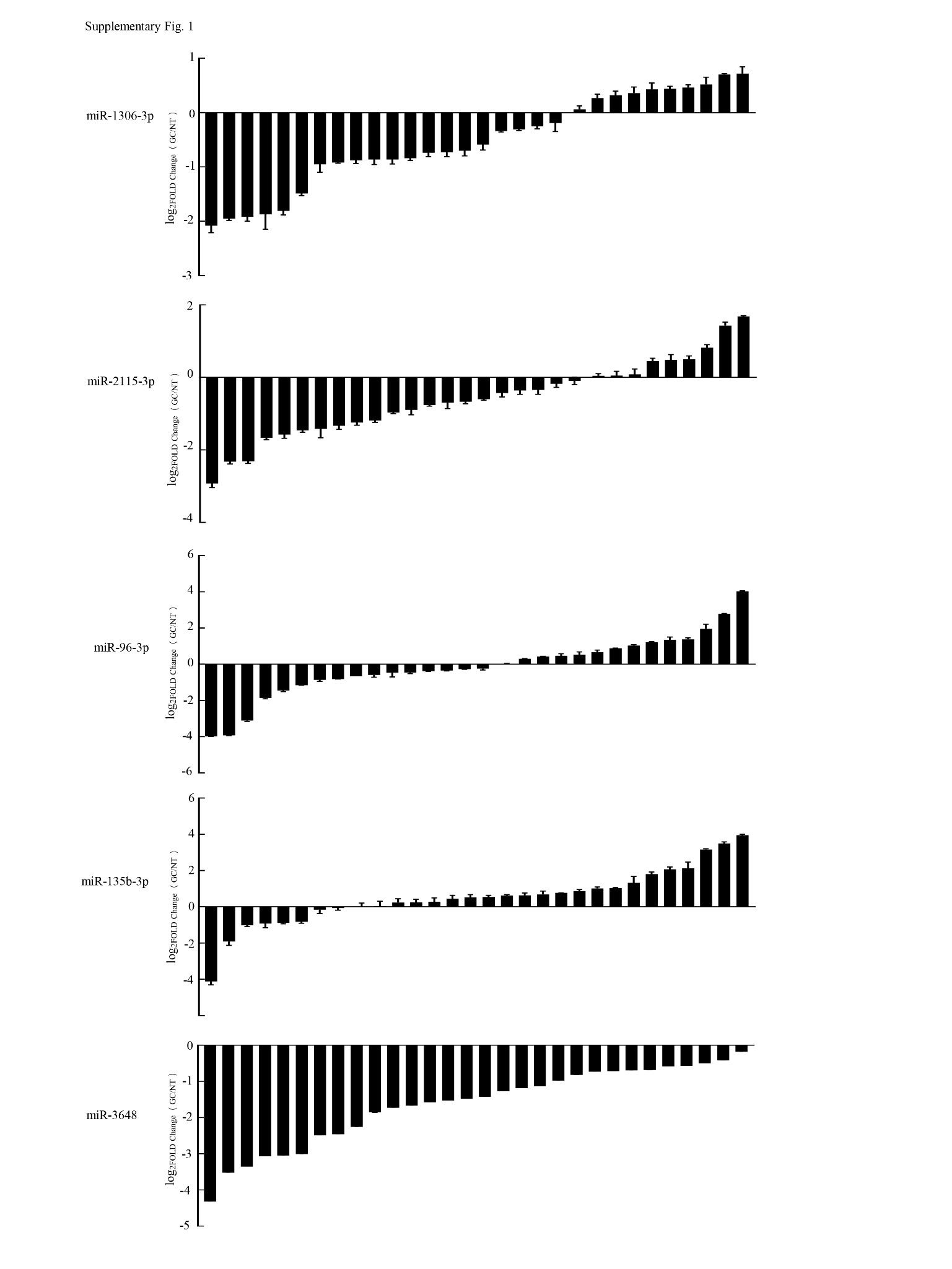


**Supplementary Fig. 1. MiR-1306-3p, miR-2115-3p, miR-96-3p, miR-135b-3p and miR-3648 expression levels in gastric tissues.** Relative miRNAs levels of GC and normal tissues measured by qPCR were shown using waterfall plot.

**
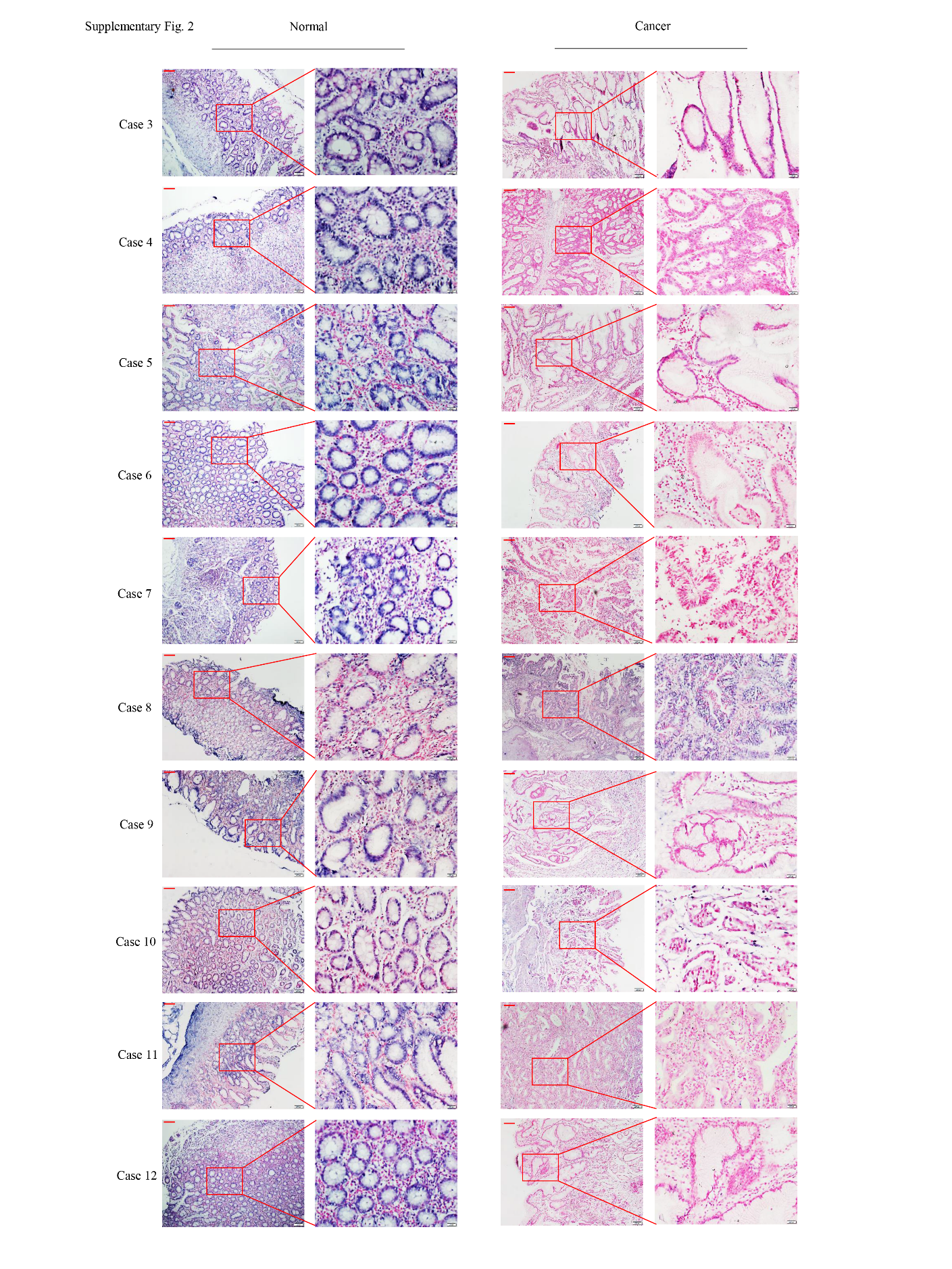
**

**Supplementary Fig. 2. The expression of miR-3648 is decreased in gastric cancer.** ISH analysis of miR-3648 expression level in normal gastric mucosa and GC tissues. Scale bars, 100 μm.


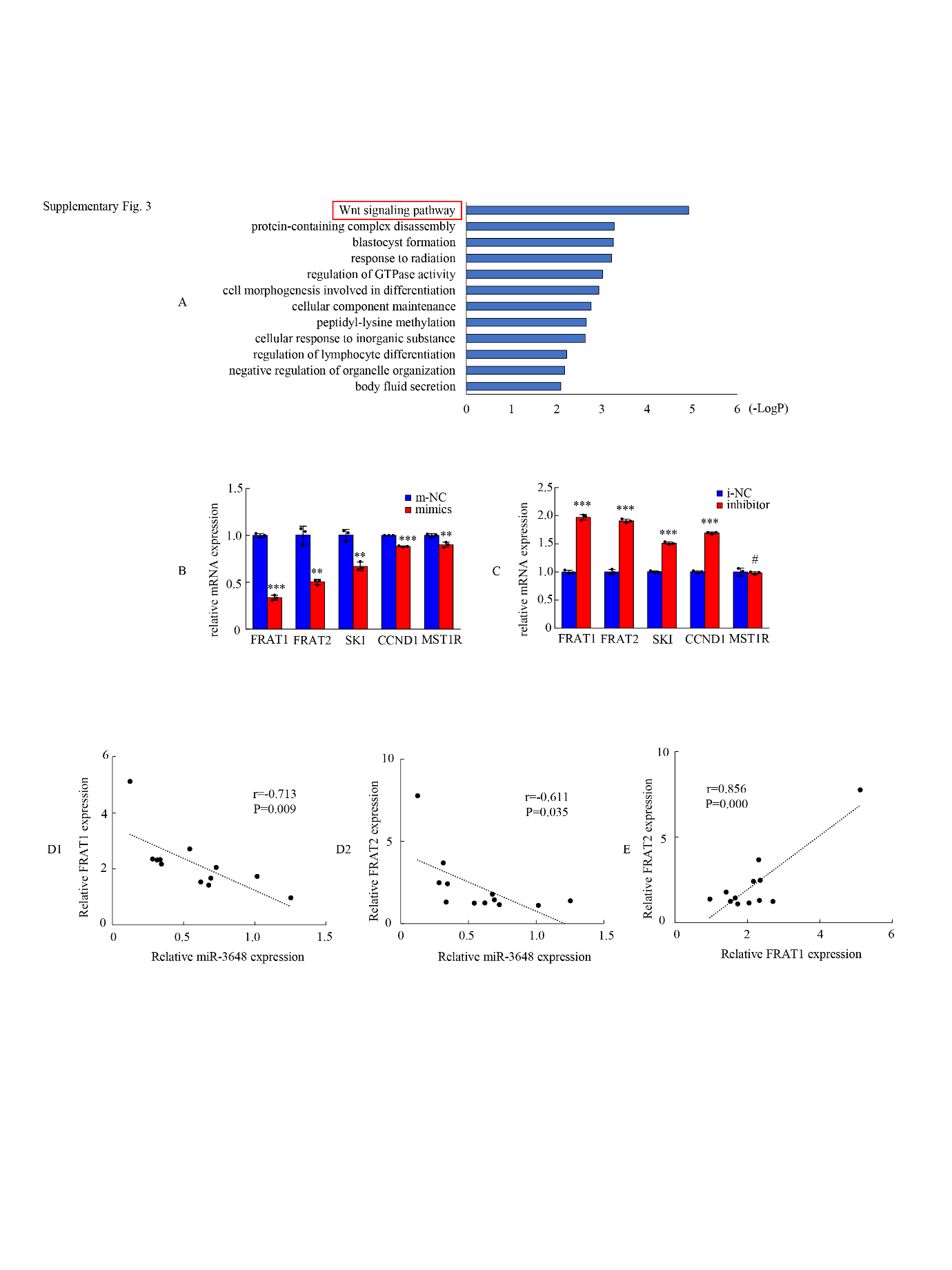


**Supplementary Fig. 3. miR-3648 is related to FRAT1/FRAT2 expression.** **(A)** Enrichment results of 102 candidate target genes in Metascape. **(B ~ C)** Changes of the expression of CCND1, SKI, FRAT1, and FRAT2 after overexpression or inhibition of miR-3648 in MKN45 or GES-1 cells (^#^*P >* 0.05, ** *P* < 0.05, *** *P* < 0.01. Two-tailed unpaired Student’s t test.). **(D1) & (D2)** In human gastric mucosal tissues, miR-3648 was negatively correlated with FRAT1 or FRAT2 expression. **(E)** FRAT1 was positively correlated with FRAT2 expression. All results were expressed as the mean of three independent experiments ± SD.


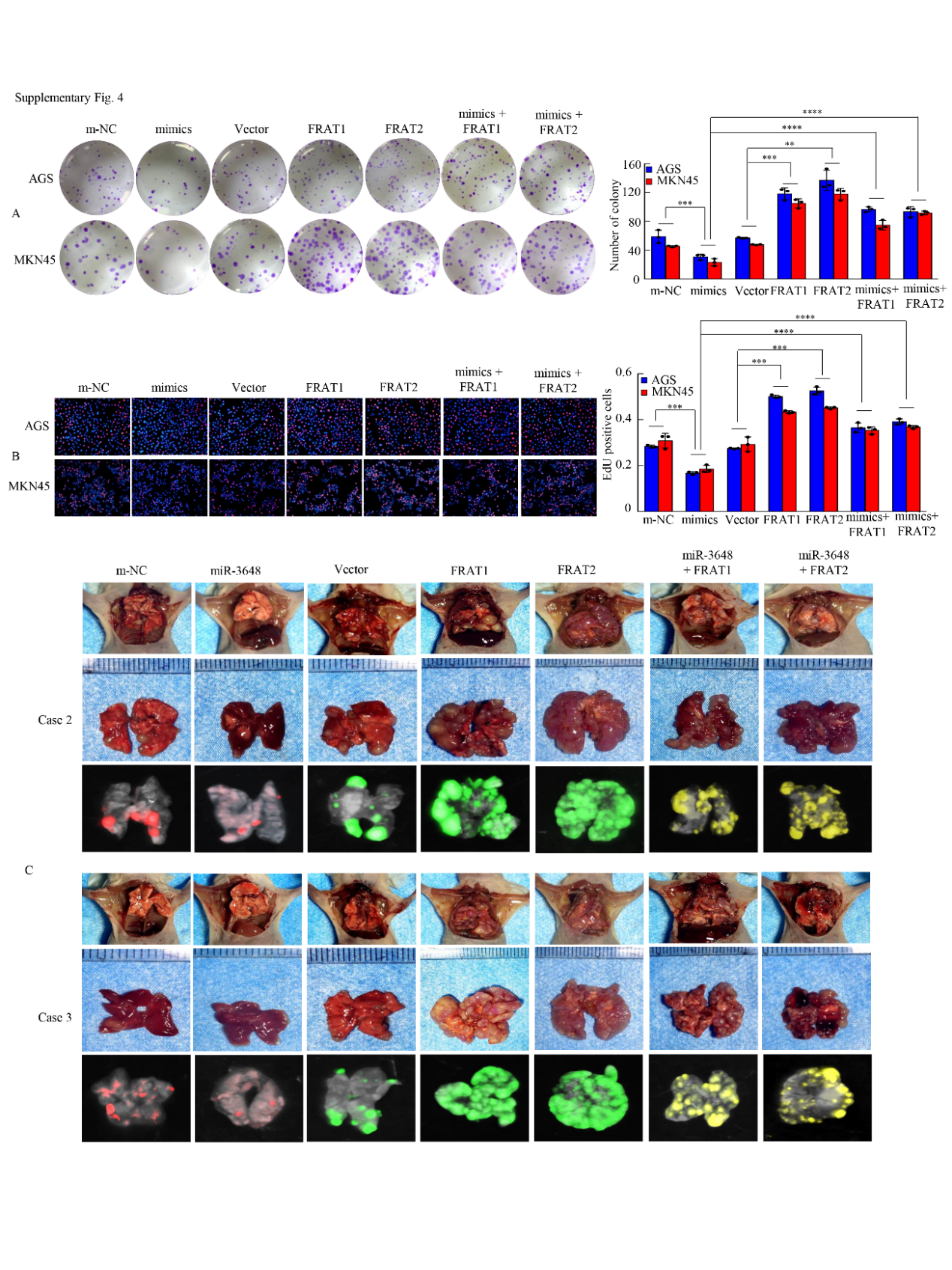


**Supplementary Fig. 4. FRAT1 or FRAT2 mediate the effects of miR-3648 on tumor proliferation and metastasis in GC cells. (A) & (B)** Restoration of FRAT1 or FRAT2 re-enhanced miR-3648-dimished proliferation in AGS and MKN-45 cells by colony formation assays and EdU. **, *P* < 0.05; ***, *P* < 0.01; ****, *P* < 0.001. Error bars represent the mean ± SD. *P* values were estimated using two-tailed unpaired Student’s t test. All experiments were repeated at least three times. **(C)** The GC MKN45 cells were orthotopically transplanted into the lung of nude mice. (n = 3 in each group, the rest 2 of 3 nude mice was showed).


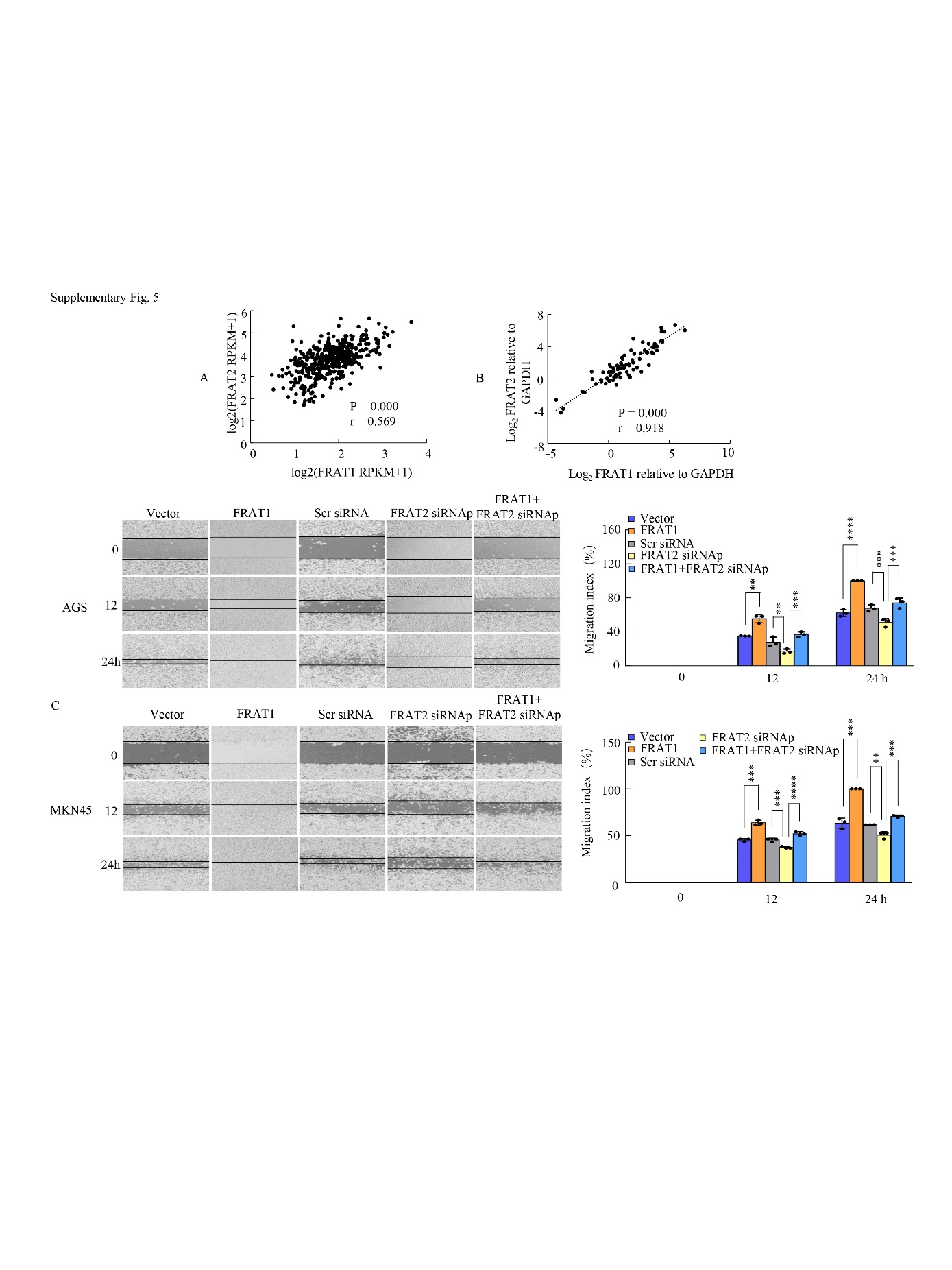


**Supplementary Fig. 5. FRAT1 synergizes with FRAT2 to promote tumour migration. (A)** The relationship between FRAT1 and FRAT2 levels was analyzed by GEPIA bioinformatics tool. **(B)** The correlation between FRAT1 and FRAT2 in 82 GC tissues using q-PCR assay. **(C)** For the wound healing experiments, cells were analyzed with live-cell microscopy.**, *P* < 0.05; ***, *P* < 0.01; ****, *P* < 0.001. Error bars represent the mean ± SD. *P* values were estimated using two-tailed unpaired Student’s t test. All experiments were repeated at least three times.


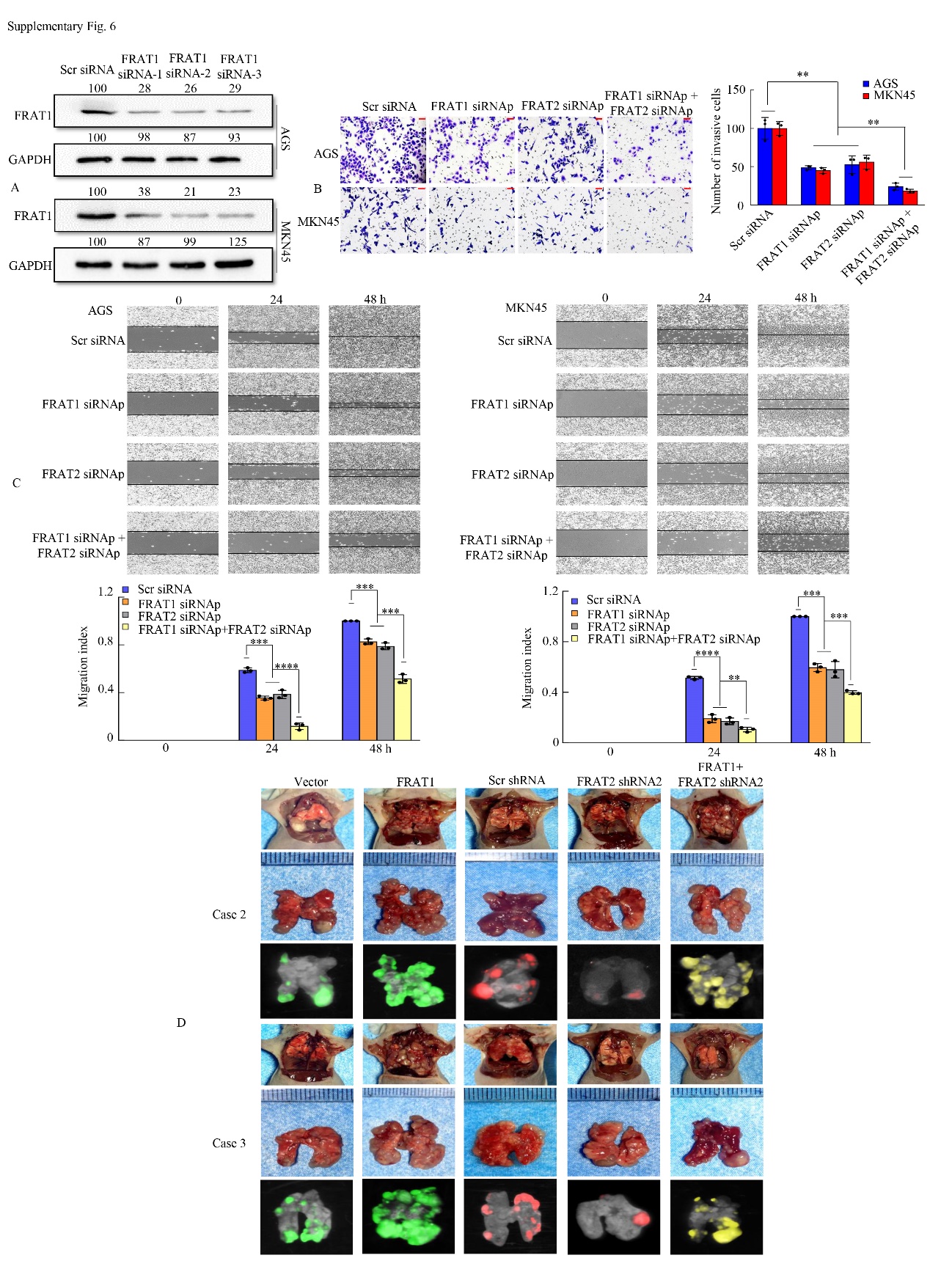


**Supplementary Fig. 6. FRAT1 synergizes with FRAT2 to promote tumour invasion and metastasis. (A)** The protein levels of FRAT1 in AGS and MKN45 cells that transfected with Scr siRNA, FRAT1 siRNA-1, FRAT1 siRNA- 2 and FRAT2 siRNA-3 detected by Western blot. **(B)** **& (C)** In vitro  the migratory and invasive ability of AGS and MKN45 cells were assessed by wound healing assay and tranwell assay. Scale bars, 50 μm in B. **, *P* < 0.05; ***, *P* < 0.01; ****, *P* < 0.001. Error bars represent the mean ± SD. Two-tailed unpaired Student’s t test was used to estimate *P* value. All experiments were repeated at least three times. **(D)** The GC MKN45 cells were orthotopically transplanted into the lung of nude mice. (n = 3 in each group, the rest 2 of 3 nude mice was showed).


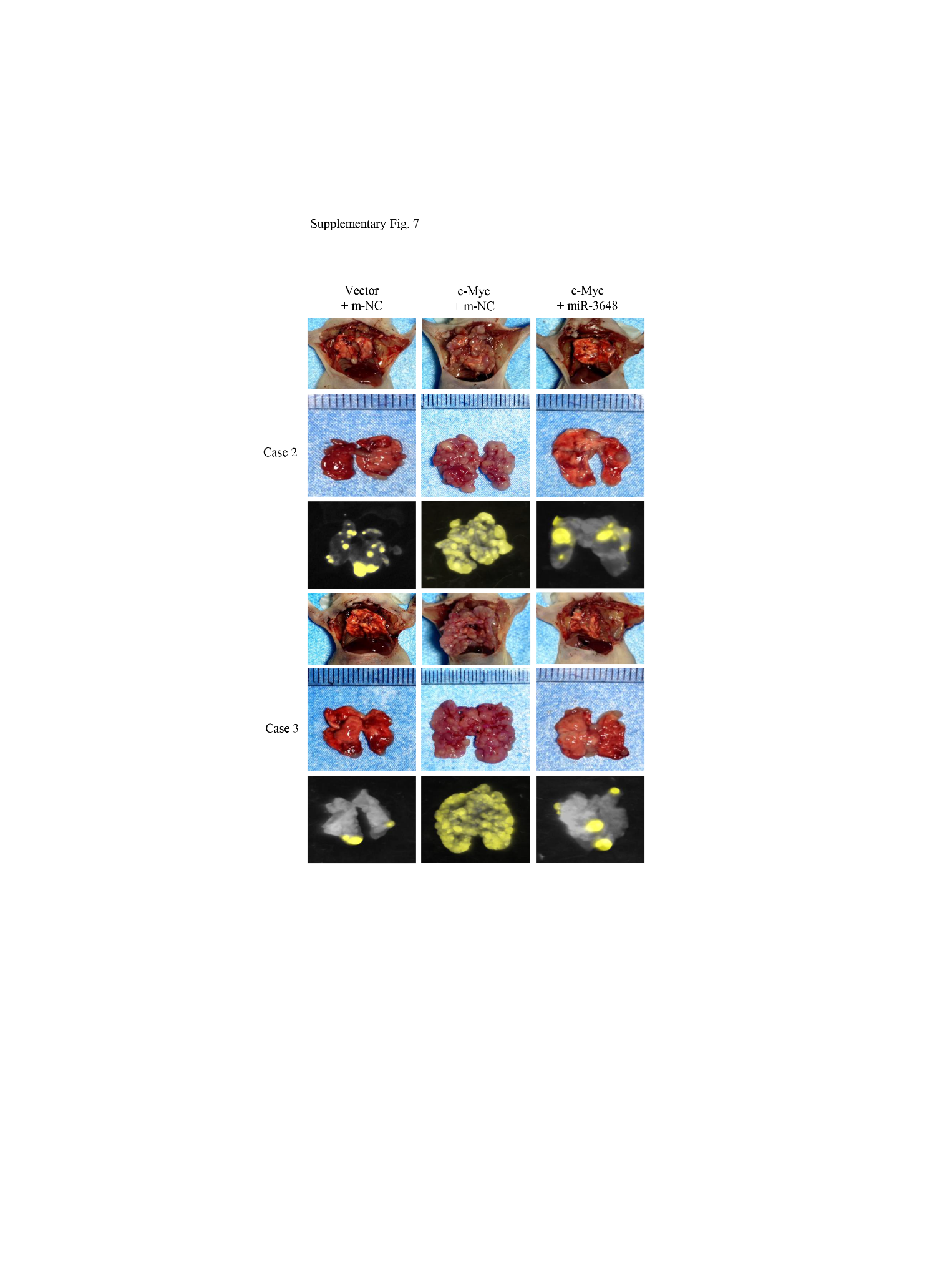


**Supplementary Fig. 7. The effect of miR-3648 on c-Myc-mediated metastasis**. The GC MKN45 cells were orthotopically transplanted into the lung of nude mice. (n = 3 in each group, the rest 2 of 3 nude mice was showed).


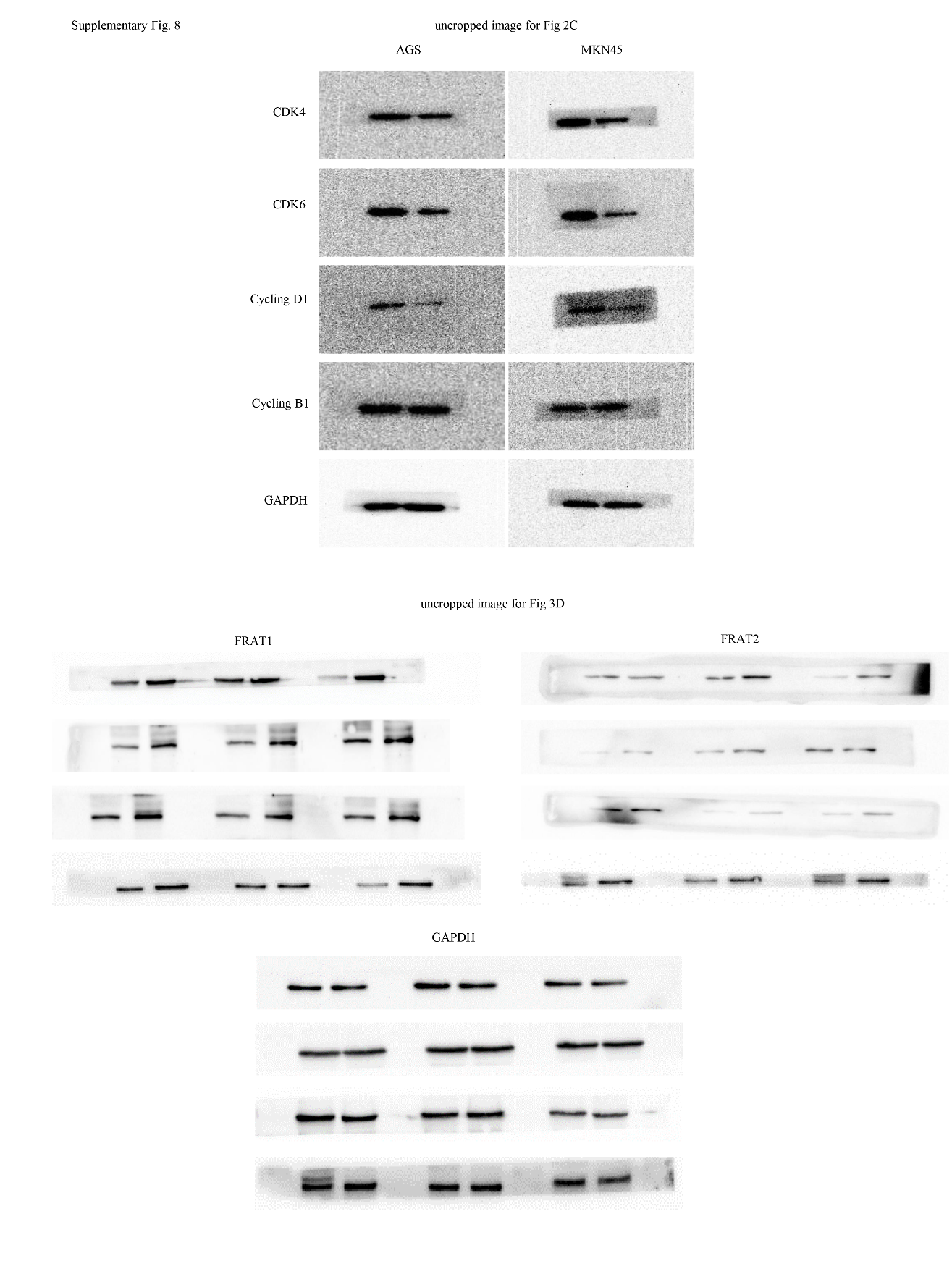


**Supplementary Fig. 8. The uncropped image of Fig. 2C & 3D.**


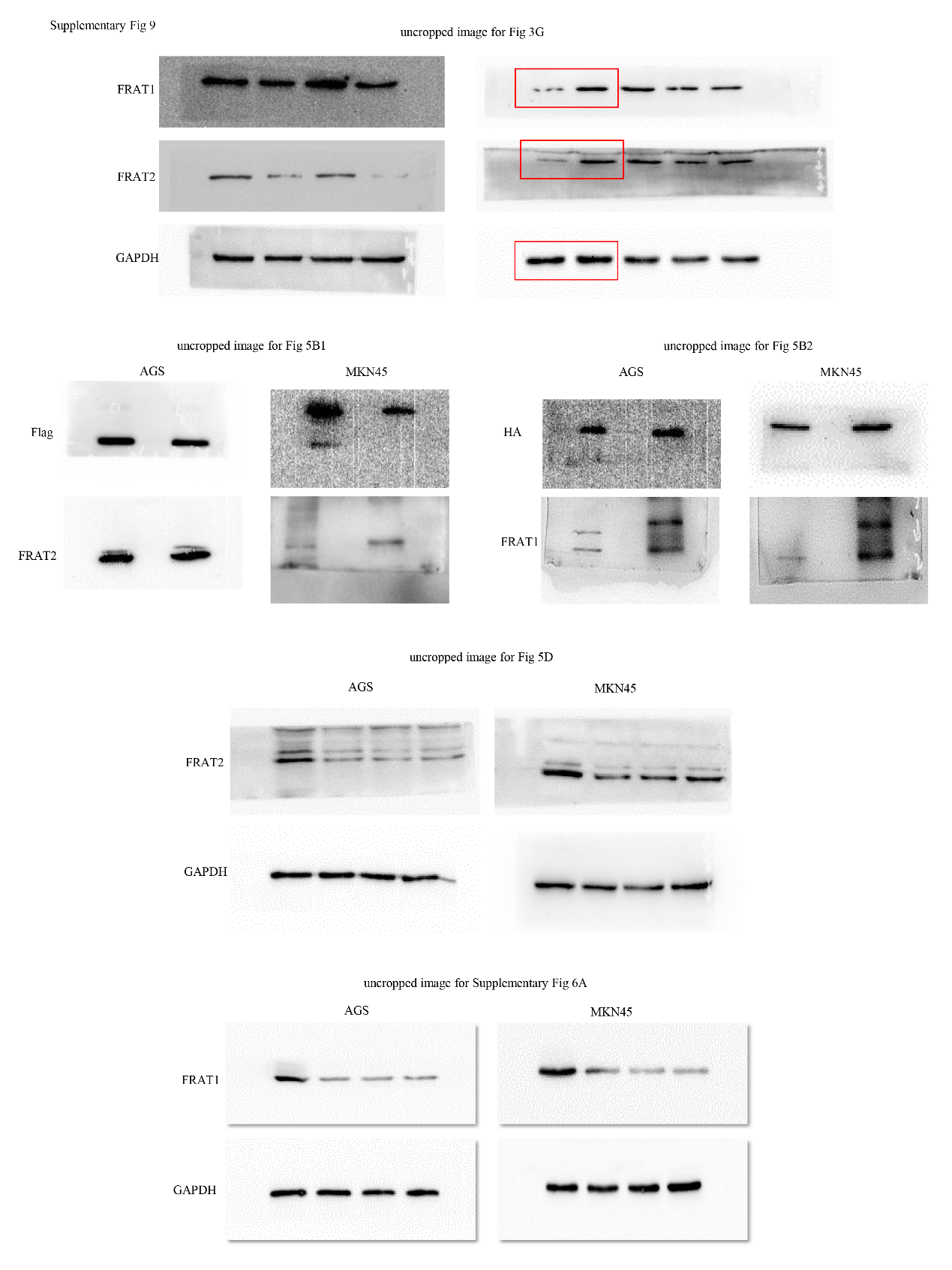


**Supplementary Fig. 9. The uncropped image of Fig. 3G , Fig. 5B, Fig. 5D and Fig. 6A.**

**
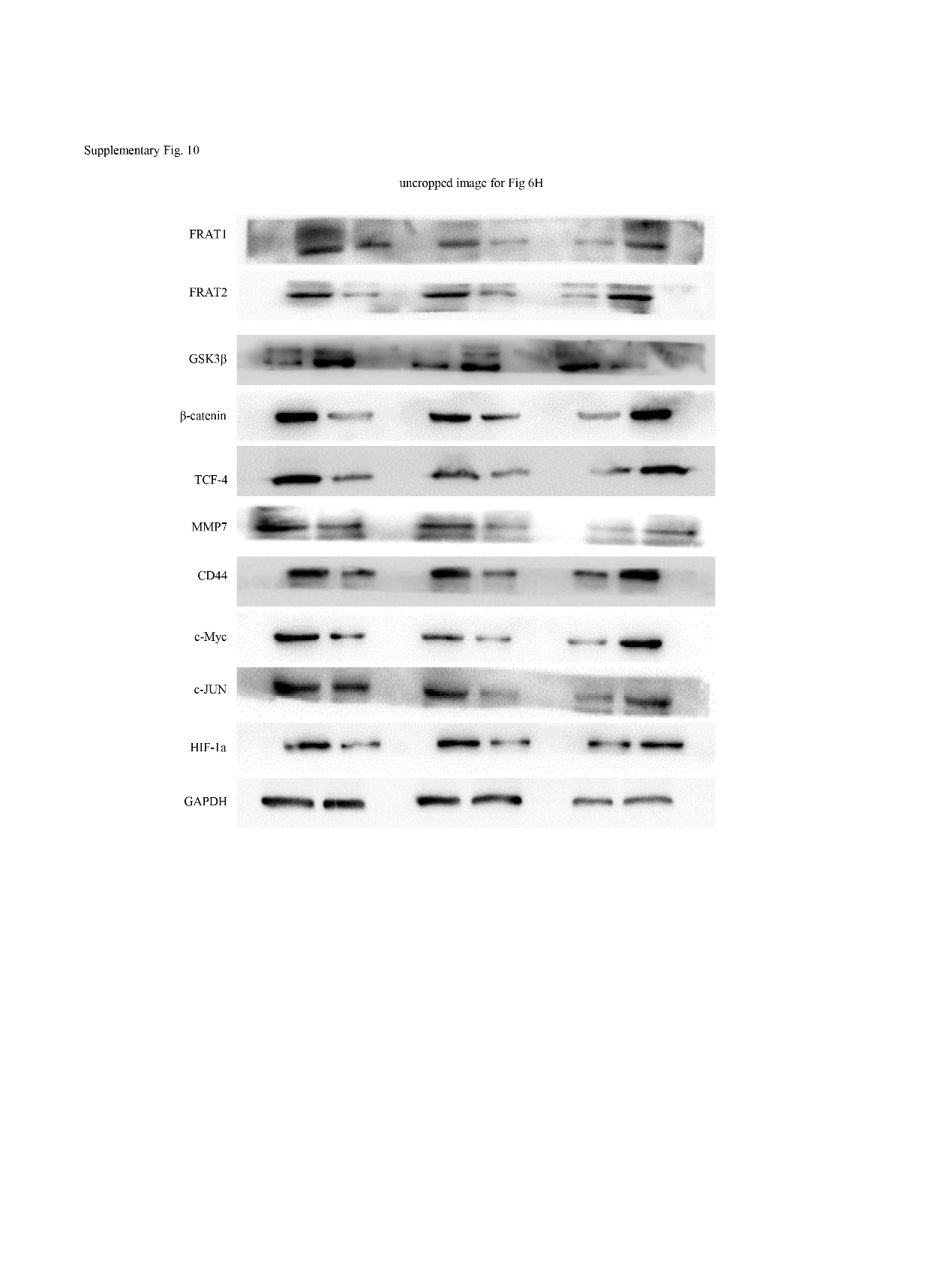
**

**Supplementary Fig. 10. The uncropped image of Fig. 6H.**
